# Deciphering the complexities of the wheat flour proteome using quantitative two-dimensional electrophoresis, three proteases and tandem mass spectrometry

**DOI:** 10.1186/1477-5956-9-10

**Published:** 2011-02-11

**Authors:** Frances M Dupont, William H Vensel, Charlene K Tanaka, William J Hurkman, Susan B Altenbach

**Affiliations:** 1 USDA Agricultural Research Service, Western Regional Research Center, Albany CA 94710, USA

## Abstract

**Background:**

Wheat flour is one of the world's major food ingredients, in part because of the unique end-use qualities conferred by the abundant glutamine- and proline-rich gluten proteins. Many wheat flour proteins also present dietary problems for consumers with celiac disease or wheat allergies. Despite the importance of these proteins it has been particularly challenging to use MS/MS to distinguish the many proteins in a flour sample and relate them to gene sequences.

**Results:**

Grain from the extensively characterized spring wheat cultivar *Triticum aestivum *'Butte 86' was milled to white flour from which proteins were extracted, then separated and quantified by 2-DE. Protein spots were identified by separate digestions with three proteases, followed by tandem mass spectrometry analysis of the peptides. The spectra were used to interrogate an improved protein sequence database and results were integrated using the Scaffold program. Inclusion of cultivar specific sequences in the database greatly improved the results, and 233 spots were identified, accounting for 93.1% of normalized spot volume. Identified proteins were assigned to 157 wheat sequences, many for proteins unique to wheat and nearly 40% from Butte 86. Alpha-gliadins accounted for 20.4% of flour protein, low molecular weight glutenin subunits 18.0%, high molecular weight glutenin subunits 17.1%, gamma-gliadins 12.2%, omega-gliadins 10.5%, amylase/protease inhibitors 4.1%, triticins 1.6%, serpins 1.6%, purinins 0.9%, farinins 0.8%, beta-amylase 0.5%, globulins 0.4%, other enzymes and factors 1.9%, and all other 3%.

**Conclusions:**

This is the first successful effort to identify the majority of abundant flour proteins for a single wheat cultivar, relate them to individual gene sequences and estimate their relative levels. Many genes for wheat flour proteins are not expressed, so this study represents further progress in describing the expressed wheat genome. Use of cultivar-specific contigs helped to overcome the difficulties of matching peptides to gene sequences for members of highly similar, rapidly evolving storage protein families. Prospects for simplifying this process for routine analyses are discussed. The ability to measure expression levels for individual flour protein genes complements information gained from efforts to sequence the wheat genome and is essential for studies of effects of environment on gene expression.

## Background

Wheat flour protein composition influences mixing and baking properties of a world commodity worth several trillion dollars annually [1,2]. Variability in flour quality is a major problem for end users but the causes are poorly understood, partly because flour contains a complex mixture of similar but distinct proteins that are difficult to separate, identify and quantify [3]. The major water-insoluble protein fraction, comprised largely of glutenin polymers and gliadin monomers, is often referred to as gluten; these proteins are also categorized among the proline- and glutamine- rich cereal storage proteins known as prolamins. High molecular weight glutenin subunits (HMW-GS) and low molecular weight glutenin subunits (LMW-GS) are linked by disulfide bonds between Cys residues to form polymers that contribute strength and elasticity to flour doughs, whereas the monomeric gliadins contribute to dough viscosity and extensibility. A single hexaploid wheat variety contains 6 genes for HMW-GS, 20 or more LMW-GS genes, 29 or more gamma-gliadins genes, up to 150 alpha-gliadin genes and at least 5 omega-gliadin genes, although not all of these genes are expressed [4-11]. In addition, some proteins with gliadin-like sequences have an odd number of Cys residues and can be linked to the glutenin polymer [7,12-16]. Flour also contains smaller amounts of other storage proteins such as globulins and triticins, proteins such as amylase and protease inhibitors that may protect against insects and fungi, and small amounts of various enzymes [17-20]. Several early studies demonstrated the utility of 2-DE to visualize the total complement of flour proteins [21,22], and the combination of 2-DE and MS techniques offers great promise for identifying these many proteins [3,23,24].

Although gliadins and glutenins are highly abundant and can be fairly well resolved by gel electrophoresis or RP-HPLC, individual proteins are difficult to identify by MS/MS. Currently, most wheat kernel or flour proteins that have been identified are from less abundant categories such as albumins and globulins [25-27]. Gliadins and glutenins were identified in 2-DE by the time consuming method of N-terminal sequencing, which is only sufficient to assign them to general categories [16,20] or by eluting individual gliadin bands from acid-PAGE and then separating the individual proteins by 2-DE to identify specific alleles [28]. Individual gliadins and glutenins have been analyzed in MS studies using combinations of fractionation, 2-DE, MS/MS, ESI/MS/MS, and MALDI [3,24,29-31], but no such study has been successful in analyzing total gliadin or glutenin composition. There are several reasons why it is difficult to precisely identify these abundant flour proteins by MS/MS. Homeologs of these proteins originated from the three genomes (A, B and D) of hexaploid bread wheat and rapid evolution gave rise to multiple gene copies including pseudogenes. Thus, the gliadin and glutenin alleles are duplicated, complex and differ significantly among wheat cultivars [8,10,14,19]. Extensive amino acid sequence coverage is needed to distinguish between these protein homeologs by MS/MS. However, the sequences are repetitive, rich in Gln and Pro, and tend to have few of the Arg and Lys residues required for digestion by trypsin, the enzyme commonly used to generate peptides for MS/MS analysis. Also, adjacent Pro residues interfere with trypsin digestion. Most gluten proteins are unique to wheat, so the complete genomic sequence databases for rice and Arabidopsis are not useful for identification of these proteins. In contrast, 80% of the proteins in a study of the wheat amyloplast proteome were identified based on similarity to orthologous proteins in the rice genome [32]. The NCBI non-redundant database contains less than 33,000 wheat gene and cDNA sequences and does not include all possible gliadin and glutenin sequences. Although over one million wheat ESTs have been arranged into provisional contigs that combine sequences from multiple cultivars, they do not represent the exact sequences for proteins of a single variety [12,13]. A proteomics method to distinguish among individual gliadins and glutenins in one variety or flour sample must overcome these difficulties [3].

Despite the lack of a method to easily identify individual LMW-GS and gliadins and relate them to specific gene sequences, a great deal is known about gluten proteins in general because of their importance as a food ingredient. Separation and identification of HMW-GS is routine, and complete genomic sequences are available for some of the most common HMW-GS gene loci [33]. The HMW-GS are encoded at *Glu-A1*, *Glu-B1 *and *Glu-D1 *on the long arms of the Group 1 homoeologous chromosomes 1A, 1B and 1D and occur as x-y pairs in close proximity. They are large, abundant proteins, ranging from 68,000 to 88,000 Daltons that are easily separated from most other flour proteins and often can be distinguished based on size alone. Because of this, significant progress has been made in relating HMW-GS alleles to flour quality [7,11,34]. Separation and identification of individual LMW-GS has been more difficult, and there has been less progress relating them to flour quality [7,35]. The classic LMW-GS are encoded at *Glu-A3*, *Glu-B3 *and *Glu-D3 *on the short arms of chromosomes 1A, 1B, and 1D, with multiple genes per locus and many allelic variants of these genes described [35-37]. The LMW-GS range in size from 32,000 to 45,000 Daltons [38].

Gamma-gliadins and omega-gliadins are encoded on the short arm of chromosome 1, with gamma-gliadins at *Gli-A1, Gli-B1*, and *Gli-D1*, and omega-gliadins at *Gli-A3, Gli-B3*, and *Gli-D3*, all of which are complex loci that contain multiple homoeologous genes, are closely linked to the *Glu-3 *LMW-GS loci, and have not yet been fully described. Gamma-gliadins are related in size and sequence to the LMW-GS [9,14,39]. Omega-gliadins tend to have molecular masses somewhat larger than the LMW-GS with sequences that consist almost entirely of repetitive motifs that differ from those of the other gluten proteins [5,6,40,41].

Alpha-gliadins are mainly encoded on the short arms of the group 6 chromosomes at *Gli-A2*, *Gli-B2 *and *Gli-D2*. They have been referred to as alpha-/beta-gliadins, because of the ability to separate them into two sub-groups by acid PAGE, but the sequences of alpha- and beta-gliadins are similar. These loci are also complex and contain multiple homoeologous genes, many of which are not expressed [8,10,42]. The alpha-gliadins range in size from 30,000 to 36,000 Daltons, overlapping in size with many gamma-gliadins and LMW-GS.

In addition to gliadins and glutenins, wheat flour contains a number of minor storage protein types, some of which are close to the traditional prolamins in composition and sequence. One minor type was given the name "avenin-like" based on similarity to oat avenins, a minor class of oat storage proteins [43]. Kasarda has proposed the name "farinins" for these avenin-like proteins because they are slightly closer in primary structure to gamma-gliadins than to avenins (D.D. Kasarda, personal communication). For similar reasons, Kasarda has proposed the name "purinins" for another type, also close to gamma-gliadins in structure and previously described as low-molecular-weight gliadins. These proteins are typified by [GenBank:ADA62372] [44]. The triticins are legumin-like 12 S globulin storage proteins encoded at *Tri-A1 *and *Tri-D1 *on the short arms of chromosomes 1A and 1D [17,45,46]. The native proteins exist as hetero-tetramers composed of long and short arms from two cleaved, disulfide-linked triticin precursors [47,48]. Several types of globulins are also detected among the flour proteins. Proteins termed globulin-1 or alpha-globulin are encoded at the highly conserved *Glo-2 *locus between the loci for the x- and y-type HMW-GS on chromosome 1 [49,50]. The type referred to as globulin-2 has similarity to known food allergens [51]. At least two copies each of globulin genes *Glo3A*, *3B *and *3D *were reported for hexaploid wheat and mapped to chromosome 4; at least one of the protein products is reported to be associated with diabetes [52].

Wheat alpha-amylase inhibitors and protease inhibitors are reported to be active against the amylases and proteases from insects such as grain-boring weevils [53]. However, they also are sufficiently abundant to serve as storage proteins for the developing grain and are a source of essential amino acids such as Lys, Met and Cys for humans who consume wheat products. The alpha-amylase inhibitors are encoded on chromosomes 3, 6 and 7 [54] and are found in monomeric (WMAI), dimeric (WDAI) and tetrameric (WTAI) forms [53]. Serpins are serine protease inhibitors found throughout the animal and plant kingdoms. The wheat serpins are suicide substrate inhibitors of chymotrypsin and cathepsin A that may serve to inactivate serine proteases of grain-boring insects [55]. They have not yet been assigned to specific genetic loci on the wheat chromosomes. Tritin is a ribosomal inhibitor [56].

Recently, methods were developed to increase the number of proteins identified by MS/MS and maximize sequence coverage for each wheat protein. Improvements included generating adequate numbers of unique peptides by digesting proteins separately with trypsin, chymotrypsin, and thermolysin, modifying MS techniques to successfully analyze the chymotryptic and thermolytic peptides, and interrogating the spectral data against a comprehensive wheat database that included sequences specific to the wheat cultivar being analyzed. Key to this approach was conducting a two pass database search, first against a large set of protein sequences and then against a subset database that also contained decoy sequences [12,13,57-59]. In this paper, these methods are applied to a quantitative 2-DE analysis of total flour protein, in order to identify as many individual flour proteins as possible and estimate their relative accumulation levels. Flour was obtained from the US hard red spring wheat Butte 86. Genes within several complex families of grain proteins have been characterized in detail in this cultivar [12,13,41,51]. Grain development, endosperm proteins and effects of environment also have been studied extensively in Butte 86 [6,25,27,32,40,60,61].

## Results

### 2-DE of total flour protein

**Figure 1:**
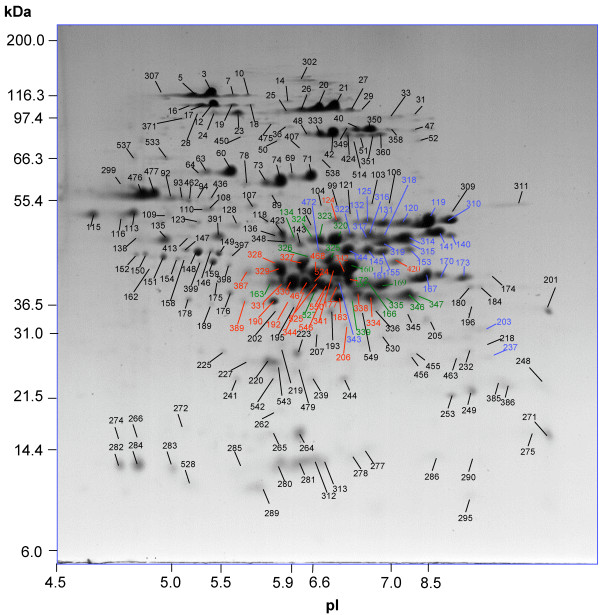
**Key to protein spot numbers in 2DE of a protein extract from white flour**. LMW-GS are labelled in blue, alpha-gliadins in red and gamma-gliadins in green. All other proteins are labelled in black. The spots are identified in Tables 3, 4, 5, 6, 7, 8, 9, 10.

2-DE resolved 476 protein spots in a total protein extract from wheat flour. Subsequently, 233 of these spots were identified by MS/MS. The identified proteins accounted for ~93% and the remaining 243 unidentified spots accounted for ~7% of the total normalized spot volume (Figure 1, Table 1). When ranked by relative spot volume, 202 of the top 250 spots were identified, including all but six spots with volumes greater than 0.1%. In addition, 25 spots were identified that were not in the top 250. The remaining unidentified spots were all minor, with spot volumes of 0.05% or less.

**Table 1:** Summary of predominant protein types identified by MS/MS in 2-DE spots of proteins from white flour of *Triticum aestivum *cv Butte 86.

Protein Type	Total # spots^1^	Total Spot Volume
HMW-GS	40	17.14
LMW-GS	29	17.99
Gamma-gliadins	16	12.17
Omega-gliadins^2^	15	10.46
Alpha-gliadins	22	20.42
Farinins	8	0.90
Purinins	6	0.82
Triticins	7	1.59
Globulins	10	0.38
GSPs and puroindolines	3	0.31
Alpha-amylase/protease inhibitors	19	4.12
Serpins	14	1.58
Other inhibitors	2	0.26
Beta-amylases	7	0.51
Other enzymes	23	1.52
Other	6	0.36
Mixed spots	6	2.56
**Total**	**233**	**93.09**

^1^Total number of spots for which the indicated protein type was the predominant protein in that spot.

^2^Spot volume includes two spots for which no omega-gliadin peptides were identified in this study; these spots were identified previously.

The Scaffold program [62] identified 168 sequences, all but seven of which were from wheat. Scaffold assigned peptides to 55 sequences from NCBI nr, 65 from large EST contig assembly databases, and 48 from Butte 86 ESTs or contigs (Table 2). The Scaffold program did not always make the most parsimonious match of peptides to protein sequences, and sometimes did not assign peptides to Butte 86 sequences that seemed to be good matches. This was in part because the database contained many redundant, incomplete, or poorly-edited sequences. Therefore a final manual evaluation of the peptide assignments was carried out as in [12,13]. After manual evaluation of the results, proteins were assigned to 157 sequences including 59 encoded by Butte 86 ESTs or contigs (Table 2, Additional files 1, 2, 3, 4, 5, 6, 7, 8, 9).

**Table 2:** 2DE-MS/MS identification of wheat flour proteins.

		Scaffold Assignments^1^				Final Assignments^2^	
			
Protein type	Total Spots^3^	Total Sequences	NCBI nr	Contig databases^4^	Butte 86 Contigs or ESTs^5^	Final Sequences	Butte 86 Contigs or ESTs^5^
HMW-GS	43	6	4	2	0	5	0
LMW-GS	35	24	9	4	11	22	10
Gamma-gliadins	34	13	2	1	10	13	10
Omega-gliadins	13	8	2	2	4	7	4
Alpha-gliadins	33	34	8	16	10	23	16
Farinins	11	3	0	0	3	3	3
Purinins	6	3	0	0	3	3	3
Triticins	9	3	0	3	0	3	0
Globulins	11	8	1	4	3	8	3
GSPs/Puroindolines	4	3	3	0	0	3	0
Alpha-amylase inhibitors	21	16	9	7	0	16	0
Serpins	15	7	2	5	0	9	6
Beta-amylases	12	4	0	1	3	4	3
Other enzymes^6^	42	30	12	18	0	33	0
Other proteins	15	6	3	2	1	5	1
**Total**		**168**	**55**	**65**	**48**	**157**	**59**

Numbers of spots and protein sequences and their database origins for each major protein category.

^1^Scaffold assignments for each spot are in Additional files 4, 5, 6, 7, 8, 9.

^2^Final assignments for each spot are in Additional file 1.

^3^Includes all 2-DE spots in which two or more peptides corresponded to sequences for the indicated protein type.

^4^Includes TaGI Releases 10.0 and 11.0, US Wheat Genome Project Assembly, HarvEST 1.14 and NCBI Unigene Build #55.

^5^Sequences derived from Butte 86 contigs are in Additional files 2, 3. Butte 86 sequences for some protein types were not analyzed in this study.

^6^Includes sequences for nine enzymes that were minor components of other spots as detailed in Additional file 1.

Single proteins were assigned to 125 spots, two proteins to 55 spots and three or more proteins to 53 spots. Peptide coverage of the identified spots ranged from 3% to 90%, with a mean of 32% and mode of 57%. In most cases the majority of the peptides from a spot were assigned to a single protein and this protein was listed as the predominant protein for that spot along with the relative spot volume, percentage coverage, and predicted molecular weight and pI (Tables 3, 4, 5, 6, 7, 8, 9, 10). Additional file 1 lists all proteins identified in each spot with the number of unique peptides and spectra for each identification. Proteins identified as Butte 86 sequences were named based on the protein type and contig number, such as LMW-GS Bu-1 or alpha-gliadin Bu-3. These names are independent of any allelic nomenclature for the proteins.

**Table 3:** Identification and quantification of HMW-GS by 2DE-MS/MS.

Spot #	Predominant protein^1^	Total proteins^2^	Spot Volume^3^	Std Dev^4^	MS/MS Coverage^5^	Predicted MW^6^	pI^7^
	HMW-GS Ax2* *(Glu-A1b-1)*						
12	HMW-GS Ax2* [GenBank:AAB02788]	1	1.22	0.024	52	86336	5.7
16	HMW-GS Ax2* [GenBank:AAB02788]	2	0.07	0.004	13	86336	5.7
17	HMW-GS Ax2* [GenBank:AAB02788]	1	0.36	0.019	34	86336	5.7
18	HMW-GS Ax2* [GenBank:AAB02788]	1	0.05	0.001	7	86336	5.7
19	HMW-GS Ax2* [GenBank:AAB02788]	1	0.14	0.005	10	86336	5.7
23	HMW-GS Ax2* [GenBank:AAB02788]	1	0.40	0.006	26	86336	5.7
24	HMW-GS Ax2* [GenBank:AAB02788]	1	0.13	0.006	22	86336	5.7
28	HMW-GS Ax2* [GenBank:AAB02788]	1	0.03	0.004	8	86336	5.7
		**Total**	**2.40**				
	**HMW-GS Bx7 ***(Glu-B1c-1)*						
20	HMW-GS Bx7 [GenBank:CAA32115]	1	1.79	0.053	56	82527	8.4
21	HMW-GS Bx7 [GenBank:CAA32115]	1	2.28	0.063	57	82527	8.4
25	HMW-GS Bx7 [GenBank:CAA32115]	1	0.12	0.026	6	82527	8.4
26	HMW-GS Bx7 [GenBank:CAA32115]	1	0.48	0.016	10	82527	8.4
27	HMW-GS Bx7 [GenBank:CAA32115]	1	0.18	0.004	5	82527	8.4
29	HMW-GS Bx7 [GenBank:CAA32115]	1	0.02	0.002	3	82527	8.4
33	HMW-GS Bx7 [GenBank:CAA32115]	1	0.05	0.002	3	82527	8.4
302	HMW-GS Bx7 [GenBank:CAA32115]	1	0.10	0.009	3	82527	8.4
		**Total**	**5.02**				
	**HMW-GS By9 ***(Glu-B1c-2)*						
40	HMW-GS By9 [GenBank:CAA43361]	2	0.38	0.003	34	73518	8.4
47	HMW-GS By9 [GenBank:CAA43361]	1	0.05	0.002	10	73518	8.4
52	HMW-GS By9 [GenBank:CAA43361]	1	0.05	0.004	19	73518	8.4
349	HMW-GS By9 [GenBank:CAA43361]	1	0.11	0.016	27	73518	8.4
350	HMW-GS By9 [GenBank:CAA43361]	1	1.32	0.004	56	73518	8.4
351	HMW-GS By9 [GenBank:CAA43361]	2	0.15	0.017	45	73518	8.4
358	HMW-GS By9 [GenBank:CAA43361]	1	0.08	0.007	20	73518	8.4
360	HMW-GS By9 [GenBank:CAA43361]	1	0.14	0.021	48	73518	8.4
		**Total**	**2.28**				
	**HMW-GS Dx5 ***(Glu-D1d-1)*						
3	HMW-GS Dx5 [GenBank:ABG68042]	1	2.37	0.020	50	87941	6.1
5	HMW-GS Dx5 [GenBank:ABG68042]	1	0.70	0.043	45	87941	6.1
7	HMW-GS Dx5 [GenBank:ABG68042]	1	0.16	0.007	3	87941	6.1
10	HMW-GS Dx5 [GenBank:ABG68042]	1	0.06	0.004	19	87941	6.1
14	HMW-GS Dx5 [GenBank:ABG68042]	1	0.05	0.007	16	87941	6.1
36	HMW-GS Dx5 [GenBank:ABG68042]	1	0.07	0.006	4	87941	6.1
138	HMW-GS Dx5 [GenBank:ABG68042]	1	0.21	0.024	4	87941	6.1
307	HMW-GS Dx5 [GenBank:ABG68042]	1	0.08	0.028	21	87941	6.1
538	HMW-GS Dx5 [GenBank:ABG68042]	1	0.05	0.002	3	87941	6.1
		**Total**	**3.75**				
	**HMW-GS Dy10 ***(Glu-D1d-2)*						
42	HMWGS Dy10 [Swiss-Prot:P10387]	2	2.32	0.051	57	67475	7.0
48	HMWGS Dy10 [Swiss-Prot:P10387]	1	0.21	0.032	57	67475	7.0
51	HMWGS Dy10 [Swiss-Prot:P10387]	2	0.17	0.014	57	67475	7.0
333	HMWGS Dy10 [Swiss-Prot:P10387]	1	0.65	0.030	57	67475	7.0
407	HMWGS Dy10 [Swiss-Prot:P10387]	1	0.07	0.019	57	67475	7.0
424	HMWGS Dy10 [Swiss-Prot:P10387]	1	0.20	0.014	57	67475	7.0
514	HMWGS Dy10 [Swiss-Prot:P10387]	1	0.07	0.010	57	67475	7.0
		**Total**	**3.69**				

Loci are indicated within parentheses.

^1^Protein that matched the greatest number of peptides detected in spot by MS/MS.

^2^Identifications of all proteins within each spot are given in Additional file 1.

^3^Normalized spot volume, average of three determinations.

^4^Standard deviation for three determinations.

^5^Percentage coverage of the mature protein by identified peptides.

^6^Predicted size of the mature protein in Daltons detemined by ExPASy ProtParam.

^7^Predicted pI for the mature protein determined by ExPASy ProtParam.

**Table 4:** Identification and quantification of LMW-GS by 2DE-MS/MS.

Spot #	Predominant protein^1^	Total proteins in spot^2^	Spot Volume^3^	Std Dev^4^	MS/MS Coverage^5^	Predicted MW^6^	pI^7^
	**LMW-GS QISQQQ-type**^8 ^*(Glu-A3f)*						
125	LMW-GS [GenBank:AAB48469]	2	0.32	0.181	41	39417	8.5
140	LMW-GS Bu4/[Swiss-Prot:P10385]	1	0.19	0.018	50	39130	8.9
141	LMW-GS Bu4/[Swiss-Prot:P10385]	1	1.10	0.031	51	39130	8.9
141a	LMW-GS Bu4/[Swiss-Prot:P10385]	2	0.58	0.059	16	39130	8.9
		**Total**	**2.19**				
	**LMW-GS QMENSHIP-type**^8 ^*(Glu-B3h)*						
314	LMW-GS Bu-2/12/13 [GenBank:ABC84366]	2	1.35	0.163	44	38153	8.2
317	LMW-GS Bu-2/12/13 [GenBank:ABC84366]	3	0.35	0.017	45	38153	8.2
318	LMW-GS Bu-2/12/13 [GenBank:ABC84366]	3	0.34	0.046	33	38153	8.2
322	LMW-GS Bu-2/12/13 [GenBank:ABC84366]	2	0.37	0.008	28	38153	8.2
119	LMW-GS Bu-3/[GenBank:BAD12055]	1	2.40	0.354	79	42589	8.5
119a	LMW-GS Bu-3/[GenBank:BAD12055]	2	0.69	0.256	62	42589	8.5
120	LMW-GS Bu-3/[GenBank:BAD12055]	1	0.78	0.112	61	42589	8.5
131	LMW-GS Bu-3/[GenBank:BAD12055]	1	0.72	0.047	48	42589	8.5
132	LMW-GS Bu-3/[GenBank:BAD12055]	1	0.10	0.008	39	42589	8.5
161	LMW-GS Bu-3/[GenBank:BAD12055]	3	<0.01	0.000	23	42589	8.5
237	LMW-GS Bu-3/[GenBank:BAD12055]	1	0.03	0.003	16	42589	8.5
310	LMW-GS Bu-3/[GenBank:BAD12055]	3	1.13	0.054	50	42589	8.5
316	LMW-GS Bu-3/[GenBank:BAD12055]	3	0.21	0.033	36	42589	8.5
		**Total**	**8.47**				
	**LMW-GS QMET-type**^8 ^*(Glu-D3a)*						
167	LMW-GS Bu-1	4	2.10	0.037	89	33008	8.7
170	LMW-GS Bu-1	3	0.33	0.021	66	33008	8.7
173	LMW-GS Bu-6	3	0.47	0.007	78	31901	8.9
144	LMW-GS Bu-7	1	1.52	0.040	50	37700	8.2
145	LMW-GS Bu-7	2	0.78	0.021	55	37700	8.2
472	LMW-GS Bu-7	3	0.17	0.071	48	37700	8.2
343	LMW-GS Bu-8	3	0.29	0.057	61	32175	7.7
203	LMW-GS Bu-11/[GenBank:AAT3786]	2	<0.01	0.000	11	39788	8.5
315	LMW-GS Bu-11/[GenBank:AAT3786]	2	0.27	0.027	52	39788	8.5
153	LMW-GS Bu-18/TC250064	1	0.26	0.030	49	37126	8.5
155	LMW-GS Bu-18/TC250064	2	0.47	0.012	50	37126	8.5
319	LMW-GS TC11_277270	2	0.67	0.038	55	39762	8.5
		**Total**	**7.33**				

Loci are indicated within parentheses.

^1^Protein that matched the greatest number of peptides detected in spot by MS/MS. Accession numbers from TaGI Release 10.0 are denoted with the prefix TC and TaGI release 11.0 with the prefix TC11_. Sequences of proteins from Butte 86 are found in Additional file 2. For partial Butte 86 contigs, the accession number for the most similar complete sequence is also indicated.

^2-7^Footnotes as in Table 3.

^8^N-terminal sequences predicted by Target P.

**Table 5:** Identification and quantification of gamma-gliadins by 2DE-MS/MS.

Spot #	Predominant protein^1^	Total proteins in spot^2^	Spot Volume^3^	Std Dev^4^	MS/MS Coverage^5,8^	Predicted MW^6,8^	pI^7,8^
325	Gamma-gliadin Bu-1 or -8	3	0.89	0.044	inc	inc	inc
326	Gamma-gliadin Bu-1	2	0.13	0.014	26	35521	7.7
160	Gamma-gliadin Bu-2	1	2.17	0.002	39	35188	8.2
166	Gamma-gliadin Bu-4^9^	3	0.41	0.024	42	32606	8.2
169	Gamma-gliadin Bu-4^9^	3	1.97	0.107	56	32606	8.2
337	Gamma-gliadin Bu-4^9^	5	0.28	0.104	63	32606	8.2
134	Gamma-gliadin Bu-5	2	0.12	0.022	37	38943	7.8
320	Gamma-gliadin Bu-5	1	2.27	0.085	50	38943	7.8
323	Gamma-gliadin Bu-5	2	0.69	0.045	56	38943	7.8
324	Gamma-gliadin Bu-5	1	0.22	0.012	55	38943	7.8
335	Gamma-gliadin Bu-6	2	0.24	0.054	47	30616	8.5
346	Gamma-gliadin Bu-6	1	1.67	0.168	49	30616	8.5
347	Gamma-gliadin Bu-6	2	<0.01	0.000	32	30616	8.5
339	Gamma-gliadin Bu-7	2	0.36	0.039	52	31010	8.2
163	Gamma-gliadin Bu-11/[GenBank:AAD30556]	1	0.38	0.037	14	32018	6.5
527	Gamma-gliadin Bu-11/[GenBank:AAD30556]	3	0.37	0.018	32	32018	6.5

^1^Protein that matched the greatest number of peptides detected in spot by MS/MS. For partial Butte 86 contigs, the accession number for the most similar complete sequence is also indicated.

^2-7^Footnotes as in Table 3.

^8^inc indicates that sequence was incomplete.

^9^Contains nine Cys residues.

**Table 6:** Identification and quantification of omega-gliadins by 2DE-MS/MS.

Spot #	Predominant protein^1^	Total proteins in spot^2^	Spot Volume^3^	Std Dev^4^	MS/MS Coverage^5,8^	Predicted MW^6,8^	pI^7,8^
	**Omega-gliadins ω-5, 1B type ***(Gli-B3h)*						
60	Omega-gliadin ω-5 type TC11_288652	1	1.39	0.126	14	50904	6.0
63	Omega-gliadin ω-5 type TC11_288652	1	0.19	0.025	13	50904	6.0
69	Omega-gliadin ω-5 type^9^	1	0.18	0.006	inc	inc	inc
71	Omega-gliadin ω-5 type TC11_288652	1	1.27	0.171	26	50904	6.0
73	Omega-gliadin ω-5 type^9^	1	0.28	0.028	inc	inc	inc
74	Omega-gliadin ω-5 type TC11-288652	1	1.77	0.235	30	50904	6.0
		**Total**	**5.08**				
	**Omega-gliadins ω-1/ω-2, 1D type **(*Gli-D3a*)						
476	Omega-gliadin *Gli-D3 *type^10^	1	2.07	0.136	inc	inc	inc
477	Omega-gliadin Bu-D1/[GenBank:AAT74547]	3	0.99	0.087	23	43525	6.0
		**Total**	**3.06**				
	**Omega-gliadins 1D **Cys type						
107	Omega-gliadin TC262770	1	0.12	0.015	8	41811	5.0
113	Omega gliadin TC262770	2	0.50	0.012	46	41811	5.0
115	Omega gliadin TC262770	2	0.47	0.065	34	41811	5.0
116	Omega gliadin TC262770	1	0.16	0.024	8	41811	5.0
		**Total**	**1.25**				
	**Omega-gliadins ω-1/ω-2, 1A type **(*Gli-A3f*)						
130	Omega-secalin-like [GenBank:ACN96903]	1	0.08	0.055	27	39492	7.0
135	Omega-gliadin Bu-D5	1	0.56	0.055	inc	inc	inc
391	Omega-gliadin Bu-D5	2	0.43	0.049	inc	inc	inc
		**Total**	**1.07**				

Loci are indicated within parentheses.

^1-7^Footnotes as in Table 4.

^8^inc indicates sequence was incomplete.

^9 ^No peptides were obtained; identified by other means.

^10 ^Beta-amylase was the predominant protein detected in spot 476 but other data indicate that this spot was predominantly omega-gliadin.

**Table 7:** Identification and quantification of alpha-gliadins by 2DE-MS/MS.

Spot #	Predominant protein^1^	Total proteins in spot^2^	Spot Vol^3^	Std Dev^4^	MS/MS Coverage^5,8^	Predicted MW^6,8^	pI^7,8^	Celiac Peptides^9^
	**Alpha-gliadins ***(Gli-A2)*							
183	Alpha-gliadin Bu-5	3	1.23	0.076	76	30506	7.1	6
344	Alpha-gliadin Bu-5	1	0.16	0.028	57	30506	7.1	6
331	Alpha-gliadin Bu-14	2	0.28	0.041	15	29995	6.2	6
190	Alpha-gliadin Bu-14	2	0.48	0.035	78	29995	6.2	6
206	Alpha-gliadin Bu-14	3	0.15	0.077	29	29995	6.2	6
546	Alpha-gliadin Bu-14	3	0.18	0.029	47	29995	6.2	6
		**Total**	**2.48**					
	**Alpha-gliadins ***(Gli-B2)*							
327	Alpha-gliadin Bu-11	5	1.59	0.107	30	34599	6.5	4
525	Alpha-gliadin Bu-12	4	0.56	0.097	65	31541	7.0	-
328	Alpha-gliadin Bu-12	4	0.27	0.058	25	31541	7.0	-
329	Alpha-gliadin Bu-12	4	1.59	0.172	27	31541	7.0	-
387	Alpha-gliadin Bu-12	4	0.17	0.017	49	31541	7.0	-
524	Alpha-gliadin Bu-12	4	0.47	0.032	80	31541	7.0	-
341	Alpha-gliadin Bu-23	3	3.29	0.154	59	33871	7.1	-
		**Total**	**7.94**					
	**Alpha-gliadins ***(Gli-D2)*							
342	Alpha-gliadin Bu-1	4	0.85	0.059	60	33412	7.8	1,2,3,4,5
330	Alpha-gliadin Bu-2^10^	6	2.66	0.148	52	30807	7.7	1,2,4
338	Alpha-gliadin Bu-2^10^	2	0.56	0.034	60	30808	7.7	1,2,4
468	Alpha-gliadin Bu-3	4	1.66	0.119	71	33156	7.0	1,2,3,4,5
467	Alpha-gliadin Bu-4	6	2.01	0.183	78	31462	6.6	1,2,3,4
550	Alpha-gliadin Bu-10	4	1.31	0.127	75	31551	6.8	1,2,3
		**Total**	**9.05**					
	**Alpha-gliadins **(unknown locus)							
124	Alpha-gliadin Bu [GenBank:BQ807130]	1	0.29	0.024	inc	inc	inc	inc
177	Alpha-gliadin Bu [GenBank:BQ806209]	5	0.32	0.118	inc	inc	inc	inc
420	Alpha-gliadin Bu-27	3	0.34	0.055	55	34268	8.3	-
		**Total**	**0.96**					
	**Gliadin mixed spots^11^**							
334	3 gamma-gliadins, 1 alpha-gliadin	4	1.08	0.105	nd	nd	nd	nd
389	1 alpha-gliadin, 1 other protein	2	0.14	0.009	nd	nd	nd	nd
172	1 gamma-gliadin, 2 alpha-gliadins	3	1.21	0.100	nd	nd	nd	nd
530	1 globulin, 1 alpha-gliadin	2	0.08	0.019	nd	nd	nd	nd
195	1 farinin, 1 alpha-gliadin, 1 enzyme	3	0.05	0.018	nd	nd	nd	nd
		**Total**	**2.56**					

Loci are indicated within parentheses.

^1-7 ^Footnotes as in Table 3.

^8^inc indicates sequence was incomplete. nd indicates not determined because the spot contained a mixture of proteins.

^9 ^The numbers indicate the presence of one or more of the following peptide sequences associated with celiac disease: 1, glia-α-9 epitope, core sequence PFPQPQLPY; 2, glia-α-20 epitope, core sequence PQPQLPYPQ; 3, glia-α-20 epitope, core sequence RPQQPYPQ; 4, glia-α epitope, core sequence QGSFQPSQQ; 5, 33-mer toxic fragment, LQLQPFPQPQLPYPQPQLPYPQPQLPYPQPQPF; 6, p31-43, LGQQQPFPPQQPY. - indicates that the alpha-gliadin does not contain any of these sequences.

^10^Contains seven Cys residues.

^11^No single protein had a predominant number of peptides. Details are in Additional file 1.

**Table 8:** Identification and quantification of farinins, purinins, triticins, globulins, and grain softness proteins by 2DE-MS/MS.

Spot #	Predominant protein^1^	Total proteins in spot^2^	Spot Volume^3^	Std Dev^4^	MS/MS Coverage^5,8^	Predicted MW^6,8^	pI^7,8^
**Farinins**							
196	Farinin Bu-1 full length	1	0.04	0.007	15	29978	8.1
385	Farinin Bu-1 C-terminus	1	0.07	0.013	13	18832	8.4
386	Farinin Bu-1 C-terminus	1	0.14	0.030	24	18832	8.4
193	Farinin Bu-2	2	0.28	0.052	38	30567	7.5
207	Farinin Bu-2	1	0.04	0.037	25	30567	7.5
549	Farinin Bu-2	1	<0.01	0.000	20	30567	7.5
336	Farinin Bu-3	2	0.33	0.043	45	30883	7.9
345	Farinin Bu-3	3	<0.01	0.000	34	30883	7.9
**Purinins**							
542	Purinin Bu-1	3	0.07	0.007	24	20272	5.9
543	Purinin Bu-1	2	0.22	0.015	38	20272	5.9
219	Purinin Bu-2	1	0.06	0.015	34	20592	6.2
223	Purinin Bu-2	1	0.20	0.030	34	20592	6.2
220	Purinin Bu-3	1	0.23	0.018	19	22371	6.2
227	Purinin Bu-3	1	0.04	0.005	28	22371	6.2
**Triticins ***(Tri-1)*							
143	Triticin [GenBank:DR736644] N-terminal subunit	3	0.27	0.011	inc	inc	inc
136	Triticin TC11_285558, N-terminal subunit	2	0.17	0.011	36	40501	6.2
348	Triticin TC11_285558, N-terminal subunit	2	0.23	0.023	36	40501	6.2
423	Triticin TC11_285558, N-terminal subunit	1	0.46	0.059	36	40501	6.2
463	Triticin TC11_285558, N-terminal subunit	1	0.06	0.003	18	40501	6.2
249	Triticin TC11_264477, C-terminal subunit	1	0.21	0.011	22	21830	8.1
253	Triticin TC11_264477, C-terminal subunit	1	0.19	0.010	17	21830	8.1
**Globulins**							
218	Globulin-1 [GenBank:ABG68030] (*Glo-2*)	1	0.05	0.023	48	22941	8.6
104	Globulin-2 Bu-17295	1	0.02	0.004	25	53832	6.6
121	Globulin-2 Bu-17295	1	0.04	0.002	23	53832	6.6
99	Globulin-2 Bu-17366	1	0.03	0.002	inc	inc	7.0
103	Globulin-2 Bu-18428	1	0.06	0.003	10	53554	6.6
106	Globulin-2 Bu-18428	1	0.03	0.004	30	53554	6.6
180	Globulin *Glo-3*-type TC234094	5	0.08	0.007	inc	inc	inc
184	Globulin *Glo-3*-type TC11_305389	2	0.03	0.008	inc	inc	inc
309	Globulin *Glo-3*-type TC11_ 305389	1	0.02	0.004	inc	inc	inc
272^9^	Globulin *Glo-3*-type TC234094/WTAI-CM3 [SwissProt: P17314]	2	0.02	0.003	nd	nd	nd
**GSP and Puroindoline ***(Pin-D1)*							
174	Grain softness protein [GenBank:CAA56591]	1	0.02	.001	17	16157	8.1
275	Grain softness protein [GenBank:CAA56586]	2	0.06	0.009	30	16381	7.6
248	Puroindoline-b [GenBank:AAT40244]	2	0.21	0.053	28	14812	9.0
271	Puroindoline-b [GenBank:AAT40244]	1	0.04	0.015	44	14812	9.0

Loci are in parentheses.

^1-7 ^Footnotes as in Table 4.

^8^inc indicates sequence was incomplete. nd indicates not determined because no single protein had a predominant number of peptides.

^9^Spot contained a similar number of peptides from two different proteins. Details are in Additional file 1.

**Table 9:** Identification and quantification of alpha-amylase and protease inhibitors by 2DE-MS/MS.

Spot #	Predominant protein^1^	Total proteins in spot^2^	Spot Volume^3^	Std Dev^4^	MS/MS Coverage^5,8^	Predicted MW^6,8^	pI^7,8^
	**Alpha-amylase and protease inhibitors**						
290	CMx1/CMx3 TC11_308146	2	0.03	0.004	57	14027	8.1
281	CMx1/CMx3 TC11_309398	3	0.23	0.029	60	13891	8.0
244	WASI [SwissProt: P16347]	1	0.17	0.027	73	19633	6.8
277	WCI [GenBank:CAD19440]	1	0.12	0.023	60	12943	7.4
283	WDAI TC11_338524	3	0.22	0.006	68	13239	5.7
286	WDAI [GenBank:AAV91972]	5	0.12	0.020	77	13191	5.2
312	WDAI [SwissProt:P01085]	3	0.67	0.125	74	13337	6.7
289	WMAI [PRF:223520]	1	0.37	0.032	72	13342	6.2
528	WMAI [PRF:223520]	1	0.13	0.022	74	13342	6.2
313	WTAI-CM1 TC11_340510	4	0.21	0.014	53	13096	6.7
285	WTAI-CM2 [SwissProt:P16851]	3	0.05	0.020	42	13034	6.2
280	WTAI-CM2 [SwissProt:P16851]	3	0.43	0.025	57	13034	6.2
264	WTAI-CM3 [SwissProt:P17314]	2	0.40	0.016	90	15832	6.7
265	WTAI-CM3 [SwissProt:P17314]	2	0.09	0.032	65	15832	6.7
266	WTAI-CM16 [SwissProt:P16159]	1	0.04	0.004	53	13437	5.0
284	WTAI-CM16 [SwissProt:P16159]	1	0.48	0.021	76	13437	5.0
274	WTAI-CM17 [GenBank:CAA42453]	1	0.02	0.004	20	13502	4.9
282	WTAI-CM17 [GenBank:CAA42453]	2	0.20	0.008	56	13502	4.9
278^9^	WCI [GenBank:CAD19440]/wheatwin-Bu-2/trypsin inhibitor factor TC11_315743	3	0.14	0.025	nd	nd	nd
	**Serpins**						
146	Serpin Bu-1 Type 1b, like [GenBank:ACN59483]	1	0.07	0.004	53	37667	5.4
147	Serpin Bu-1 Type 1b, like [GenBank:ACN59483]	1	0.09	0.006	64	37667	5.4
148	Serpin Bu-1 Type 1b, like [GenBank:ACN59483]	1	0.44	0.015	57	37667	5.4
158	Serpin Bu-1 or Bu-4	1	0.02	0.011	inc	inc	inc
162	Serpin Bu-1 or Bu-4	1	0.02	0.005	inc	inc	inc
149	Serpin Bu-2 Serpin Z1c, like [SwissProt:Q9ST58]	2	0.17	0.012	70	42882	5.6
397	Serpin Bu-2 Serpin Z1c, like [SwissProt:Q9ST58]	1	0.02	0.004	18	42882	5.6
398	Serpin Bu-3, Z1a type [Swiss-Prot: P93693]	1	0.04	0.006	40	43118	5.6
399	Serpin Bu-3, Z1a type [Swiss-Prot: P93693]	1	0.04	0.007	24	43118	5.6
159	Serpin Bu-4 or Bu-5	3	0.24	0.007	inc	inc	inc
151	Serpin Bu-5, like [GenBank: CAA72274]	1	0.19	0.016	62	42981	5.2
154	Serpin Bu-5, like [GenBank: CAA72274]	1	0.08	0.011	23	42981	5.2
150	Serpin Bu-7, like [GenBank:ACN59484]	1	0.10	0.010	62	43431	5.1
152	Serpin Bu-7, like [GenBank:ACN59484]	1	0.06	0.016	39	43431	5.1
	**Other Inhibitors**						
201	Tritin TC235992	4	0.15	0.017	64	29653	9.8
205	Xylanase inhibitor XIP-1 [PDB:1OM0]	1	0.11	0.042	54	30285	8.3

^1-7 ^Footnotes as in Table 4.

^8 ^inc indicates the sequence was incomplete. nd indicates not determined because no single protein had a predominant number of peptides.

^9 ^Spot contained a similar number of peptides from three different inhibitors. Details are in Additional file 1.

**Table 10:** Identification and quantification of enzymes and other factors by 2DE-MS/MS.

Spot #	Predominant protein^1^	Total proteins in spot^2^	Spot Volume^3^	Std Dev^4^	MS/MS Coverage^5^	Predicted MW^6^	pI^7^
	**Beta-amylase ***(β-Amy-A1*, β-Amy-B1, β-Amy-D1)						
476	Beta-amylase Bu-1^8^	4	-	-	32	60016	6.9
537	Beta-amylase Bu-1	2	0.04	0.010	18	60016	6.9
94	Beta-amylase Bu2	2	0.04	0.006	47	54481	5.9
462	Beta-amylase Bu2	2	0.11	0.014	60	54481	5.9
64	Beta-amylase Bu3	1	0.05	0.010	13	54319	5.8
92	Beta-amylase Bu3	1	0.18	0.029	34	54319	5.8
93	Beta-amylase Bu3	1	0.09	0.013	55	54319	5.8
	**Other enzymes^9^**						
89	ADP-glucose PP lg subunit [GenBank:CAD98749]	1	0.03	0.002	10	53030	5.6
128	ADP-glucose PP lg subunit [GenBank:CAD98749]	1	0.10	0.047	11	53030	5.6
108	ADP-glucose PP sm subunit [GenBank:AAF61173]	2	0.09	0.013	60	52061	5.5
110	ADP-glucose PP sm subunit [GenBank:AAF61173]	2	0.06	0.019	24	52061	5.5
118	Alanine amino transferase TC11_282456	1	0.06	0.002	36	52820	6.1
109	ATP-synthase beta-subunit [GenBank:CAA52636]	1	0.02	0.004	32	58562	5.4
232	Chitinase, rye, [GenBank:BAB18520]	1	0.04	0.009	40	26095	8.7
455	Chitinase [GenBank:AAX83262]	1	0.05	0.010	26	26022	8.3
241	Dehydroascorbate reductase TC264934	2	0.09	0.013	83	23358	5.9
123	Enolase TC11_292359	2	0.07	0.014	64	48033	5.4
178	Glyoxalase I TC11_288238	1	0.10	0.020	68	32568	5.4
202	Glucose/ribitol dehydrogenase RS_UWI_14903	1	<0.01	0.000	57	31851	6.3
436	Ketol-acid reducto isomerase TC234371	3	0.05	0.005	21	57486	5.4
175	Malate dehydrogenase [GenBank:AAT64932]	1	0.03	0.007	41	35486	5.8
176	Malate dehydrogenase [GenBank:AAT64932]	1	0.15	0.000	58	35486	5.8
450	Methionine synthase RS_UWI_10957	1	0.02	0.005	11	84552	5.7
371	Orthophosphate dikinase TC11_322894	1	0.03	0.004	19	73501	5.8
299	PDI3 [GenBank:AAK49425]	3	0.14	0.016	53	54094	4.9
475	Sucrose synthase 2 [GenBank:CAA03935]	1	0.08	0.011	19	92608	6.2
189	Thiamine biosynth enzyme TC11_308909	2	0.08	0.007	35	33167	5.7
225	Triose-phosphate isomerase [GenBank:CAC14917]	1	0.05	0.006	38	26803	5.4
239	27 K thiol reductase-like TC11_300123	1	0.16	0.037	54	23642	6.1
479	27 K thiol reductase like TC11_299048	2	0.02	0.005	16	23788	6.1
	**Other**						
311	Elongation factor EF1A [Swiss-Prot: Q03033]	1	0.11	0.005	14	49169	9.2
533	HSP70 (Butte 86) [GenBank:AAB99745]	1	0.08	0.009	18	71031	5.1
413	Initiation factor Eif4A [Swiss-Prot: P41378.1]	1	0.06	0.014	36	46928	5.3
295	LTP Bu-2	1	0.04	0.009	38	9606	8.2
456	Thaumatin-like protein TC11_283136	2	0.05	0.007	39	21408	7.9

Loci are indicated within parentheses if known.

^1 ^Protein that matched the greatest number of peptides detected in spot by MS/MS. Accession numbers from TaGI Release 10.0 are denoted with the prefix TC and TaGI release 11.0 with the prefix TC11_. Accession numbers from HarvEST 1.14 are denoted with RS_UWI_. Sequences of proteins from Butte 86 are found in Additional file 2.

^2-7 ^Footnotes as in Table 3.

^8^Although Scaffold identified more peptides for beta-amylase in spot 476, other data indicate that this spot was predominantly omega-gliadin.

^9^The following enzymes or factors were detected as minor components of other spots: alcohol dehydrogenase, aspartic protease, formate dehydrogenase, fructose bisphosphate aldolase, GDP-binding protein, manganese superoxide dismutase, peroxidase, peroxiredoxin, phosphoglycerate kinase.

### HMW-GS

HMW-GS were identified in 43 spots and were the primary protein identified in 40 spots that accounted for 17% of total spot volume (Figure 1, Tables 1, 2, 3, Additional file 1). These are clustered near the 116,300 Dalton marker in the center of the gel, at higher apparent molecular weights and more acidic pIs than predicted from the protein sequences. Scaffold assigned peptides to six HMW-GS sequences, with four from NCBI nr and two from contig assembly databases (Table 2). After manual evaluation of the data the peptides were assigned to five HMW-GS sequences based on gene sequences from the cultivar Cheyenne (Table 2, 3, Additional file 1). These five HMW-GS types are consistent with reports that the *Glu-A1*, *Glu-B1 *and *Glu-D1 *loci each encode only one x-type and one y-type HMW-GS, with *Glu-A1y *generally not expressed. In Butte 86, the five HMW-GS proteins and their loci were Ax2* [GenBank:AAB02788] *(Glu-A1-1c)*, Bx7 [GenBank:CAA32115] *(Glu-B1-1a)*, By9 [GenBank:CAA43361] *(Glu-B1-2b)*, Dx5 [GenBank:ABG68042] *(Glu-D1-1d) *and Dy10 [Swiss-Prot:P10387] *(Glu-D1-2b)*. The HMW-GS formed charge trains in 2-DE, with an average of eight spots per protein. These were Ax2* (12, 16, 17, 18, 19, 23, 24, 28); Bx7 (20, 21, 25, 26, 27, 29, 33, 302); By9 (40, 47, 52, 349, 350, 351, 358, 360); Dx5 (3, 5, 7, 10, 14, 36, 138, 307, 538); and Dy10 (42, 48, 51, 333, 407, 424, 514). Ax2* and Dx5 resolved at more acidic pIs than Bx7, By9 and Dy10. A few minor spots were resolved at slightly different apparent molecular weights from the primary charge trains and could result from proteolysis or post-translational modification. Spots identified as Ax2* (23, 24, 28), Dx5 (14, 36), Bx7 (33), By9 (52, 351, 360) or Dy10 (51, 407, 424) resolved at slightly lower molecular weights. Spots identified as Dx5 (138, 158) resolved at considerably lower molecular weights and a spot identified as Bx7 (302) resolved at a higher molecular weight. Amino acid sequence coverages of 50% to 57% were obtained for the largest spots. HMW-GS were the only type of protein identified in the HMW-GS spots and cross-contamination between HMW-GS types was detected only for four minor spots with a mix of By9 and Dy10 peptides (40, 42, 51, 351) and one Ax2* spot with traces of Bx7 (16). The most abundant HMW-GS type was Bx7, which is highly expressed in many varieties. Bx7 accounted for 5.0% of total spot volume and 29.3% of the HMW-GS. The gene for Bx7 is reported to be duplicated in some varieties, but this has not been reported for the Cheyenne type Bx7 allele found in Butte 86.

### LMW-GS

**Figure 2:**
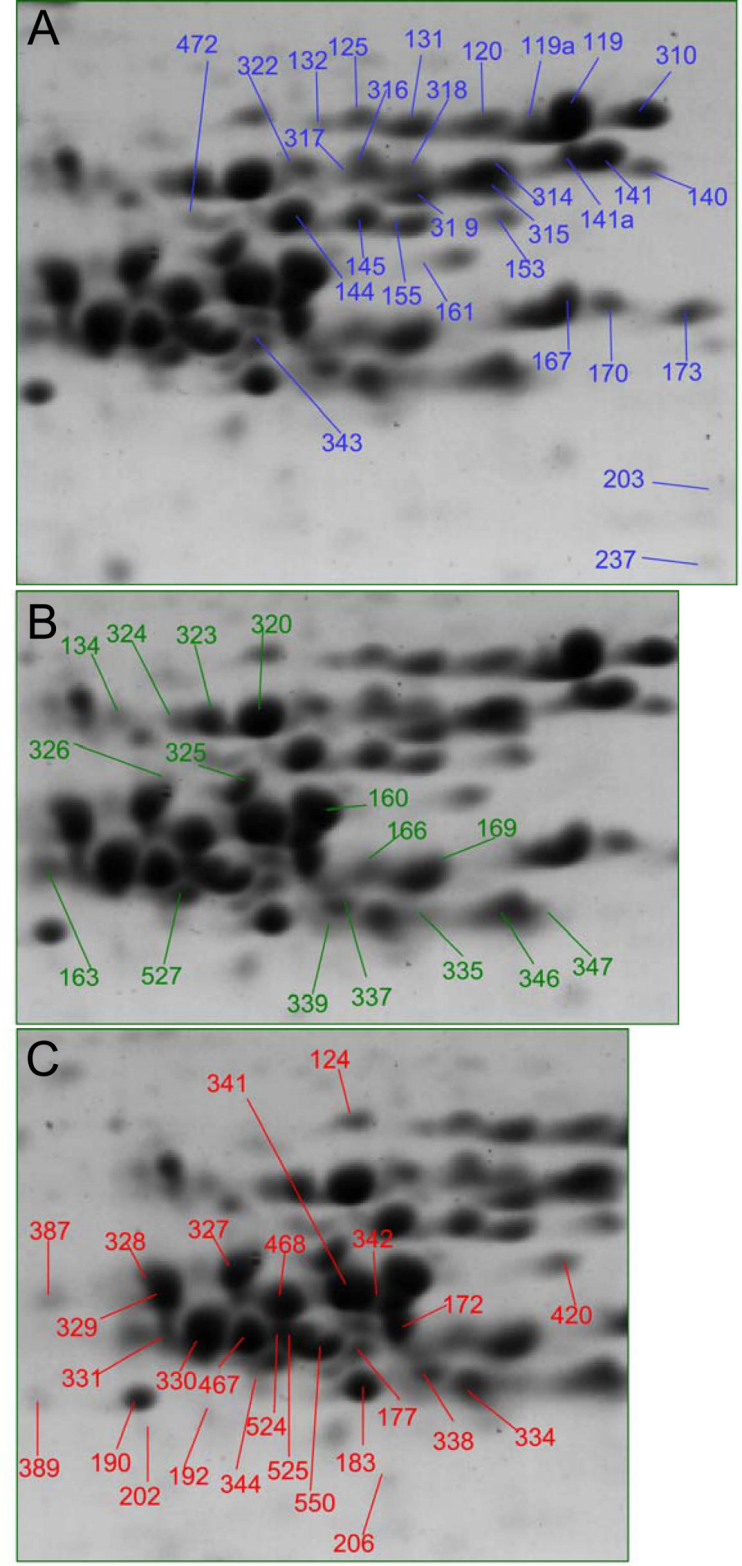
**Key to spot numbers for LMW-GS, alpha-gliadins and gamma-gliadins in 2DE of a protein extract from white flour**. A, Spots for which the only or predominant proteins identified were LMW-GS; B, gamma-gliadins; and C, alpha-gliadins. The spots are identified in Tables 4, 5 and 6.

LMW-GS were identified in 35 spots and were the primary protein identified in 29 spots that accounted for 18% of total flour protein (Figures 1, 2A; Tables 1, 2, 4; Additional file 1). Nine of these spots also contained at least two peptides from another protein type, such as a peroxidase, alpha-gliadin or gamma-gliadin (Additional file 1). The proteins, labeled in blue, were resolved in the center of the gel, at the more basic end of a dense cluster of LMW-GS, gamma-gliadins, and alpha-gliadins. All but two proteins were resolved between the 36,500 and 55,400 Dalton markers and at pIs from 6.5 to 8.5, higher molecular weights and more acidic pIs than predicted based on the protein sequences. Scaffold assigned the peptides from the 35 spots to 24 different LMW-GS sequences, including nine from NCBI, four from large contig databases and 11 encoded by Butte 86 contigs (Table 2). After manual evaluation of the results, the peptides were reassigned to 22 distinct LMW-GS sequences including ten Butte 86 LMW-GS contigs (Table 2). Association of LMW-GS with sequences from Butte 86 was complicated by the fact that many of the Butte 86 contigs were missing coding sequences for the N-terminal portions of the proteins. Complete sequences from NCBI nr that matched the Butte 86 contigs are indicated (Table 4, Additional file 1). Amino acid sequence coverages of 11 to 89% were obtained for the predominant proteins in each spot (Table 4).

The Butte 86 LMW-GS proteins matched the *Glu-A3f*, *B3h*, *D3a *allele pattern in 1-D SDS-PAGE and 2-DE (Ikeda and Pena, personal communication), so the following are based on these probable assignments. LMW-GS were originally characterized based on N-terminal amino acid sequences. Because there was evidence for modification of the N-termini, the predicted (Table 4) and observed N-termini of each LMW-GS (Additional file 1) are noted.

#### LMW-GS *Glu-A3*

Peptides from four spots matched LMW-GS sequences with predicted N-terminal sequences beginning with QISQQQQ (Table 4). This type of LMW-GS is reported to be associated with the *Glu-A3 *locus. LMW-GS Bu-4, lacking the N-terminus, and the similar complete sequence [Swiss-Prot:P10385] accounted for all of the peptides in two adjacent spots (140, 141) and the majority in a third spot (141a) to the basic side of the LMW-GS cluster (Figure 2A). Together they amounted to 1.9% of total spot volume and 10.4% of the total LMW-GS. Spot 141a included an N-terminal peptide beginning with QISQQQ (Additional file 1). Peptides from spot 125 matched a similar but distinct sequence, [GenBank:AAB48469]. Spot 125 was located above and to the left of the 140, 141, 141a group, among spots attributed to the *Glu-B3 *allele.

#### LMW-GS *Glu-B3*

Peptides from 17 spots matched LMW-GS sequences with predicted N-terminal sequences beginning with QMENSHIP (Additional file 1). LMW-GS of this type were the predominant protein in 13 spots (Table 4) and are reported to be associated with the *Glu-B3 *locus. Scaffold assigned the peptides to eight separate LMW-GS sequences. However, further evaluation of the data indicated that the peptide data could be accounted for by five sequences (Additional file 1).

The contig assembly LMW-GS Bu-3 encodes a partial protein sequence that matched the complete sequence [GenBank:BAD12055] with a predicted N-terminal sequence of QMENSHIP, size of 42,589 Daltons and pI of 8.5. It was identified as the major component of five adjacent spots (119, 120, 131, 132, 310) comprising a likely charge train at the top of the LMW-GS cluster, two spots (161, 316) of a lesser apparent size, and spot 237, a putative fragment, and was a minor component of three other spots (119a, 125, 317). This is a highly expressed protein, contributing 6.1% of total spot volume and 33.7% of the total LMW-GS. A peptide beginning with the predicted N-terminus QMENSHIP was identified only in spot 125, at the acidic end of the peptide group, whereas peptides beginning with the alternative N-terminus SHIP were identified in six of the spots (119, 119a, 120, 131, 161, 310) (Additional file 1). Scaffold assigned these N-terminal peptides to a similar sequence [GenBank:CAB450553] that began SHIP because the parameters chosen for analysis of the data did not allow unusual cleavages. However, all peptides could be accounted for by LMW-GS Bu-3/[GenBank:BAD12055] except for a single peptide in spot 119a that was unique to [GenBank:CAB450553]. Two peptides from spot 310 were assigned to the similar sequence [GenBank:BAB78741].

Peptides from the predominant protein in four spots (314, 317, 318, 322) were assigned to three similar but incomplete contigs, LMW-GS Bu-2, Bu-12 and Bu-13. The three contigs were distinct but the peptides could not be uniquely assigned to single contigs. The C-terminal halves of the sequences for Bu-2, Bu-12 and Bu-13 encoded identical protein sequences and the N-terminal sequence of each contig was based on a single EST. The N-terminal coding regions of Bu-2 and Bu-12 were missing and Bu-13 may be missing a portion of the internal sequence. The complete NCBI sequence [GenBank:ABC84366] matched the last 172 amino acids of LMW-GS Bu-2, Bu-12 and Bu-13 and encodes a protein of 38,153 Daltons, pI of 8.2 and N-terminus of QMENSHIP. [GenBank:ABC84366] was used to estimate physical parameters for these proteins. A peptide beginning SHIP was identified for spot 314 (Additional file 1).

Peptides matching the contig TC11_277260 were minor components of a cluster of four spots (141a, 314, 317, 319) near and within the LMW-GS Bu-2/-12/-13 group (Figure 2A, Additional file 1). TC11_277260 did not match any Butte 86 LMW-GS contigs or any complete sequence from NCBI nr, and the ESTs that comprised the contig were assigned to four distinct new contigs in a later version of the TaGI contig assembly database (DFCI TaGI Release 12.0) [63]. Therefore, the exact sequence for the proteins in these spots is not yet known.

In all, the 13 spots that were primarily QMENSHIP-type LMW-GS accounted for 8.5% of total spot volume, or 47.1% of total LMW-GS (Table 4). Additional DNA sequences and further analysis are needed to determine the exact number of proteins in this complex allelic group.

#### LMW-GS *Glu-D3*

Peptides from 12 spots were assigned to LMW-GS with predicted N-terminal sequences beginning QMET (Table 4, Additional file 1). LMW-GS of this type are reported to be associated with *Glu-D3*. Scaffold assigned the peptides to nine different gene sequences. The 12 spots accounted for 7.3% of total flour protein and 40.7% of total LMW-GS.

LMW-GS Bu-1 was the major protein in two adjacent spots at the lower right of the LMW-GS cluster (167, 170) accounting for 2.4% of total spot volume (Figure 2A, Table 4). It had a predicted N-terminal sequence beginning QMETRCIP. N-terminal peptides were not detected in either spot. Both spots also contained peptides for peroxidase, which resolves in the same area (Additional file 1). LMW-GS Bu-7 with a predicted N-terminal sequence of QMETSRV accounted for the majority of the peptides in three adjacent spots in the middle left of the LMW-GS cluster (144, 145, 472) accounting for 2.5% of total spot volume. An N-terminal peptide beginning QMETSRVP was identified for spot 472 and a peptide beginning METSRV was identified for spot 145 (Additional file 1). Peptides corresponding to this LMW-GS were also minor components of eight other spots. LMW-GS Bu-6 with a predicted N-terminal sequence of QMETSCIP was the predominant protein in spot 173 along with a peroxidase, at the lower right of the LMW-GS cluster. LMW-GS Bu-8 with a predicted N-terminal sequence of QMETSCIS was the primary protein in spot 343, at the lower left of the LMW-GS. Peptides from four spots were assigned to three different sequences beginning QMETSHIP. Peptides from spots 153 and 155 were assigned to TC250064, which is similar to LMW-GS Bu-18, and the predominant protein in spots 203 and 315 was [GenBank:AAT37861] which is similar to LMW-GS Bu-11. Spot 203 is a minor protein that may be a LMW-GS fragment.

### Gamma-gliadins

Gamma-gliadins were identified in 34 spots. Sixteen spots contained primarily gamma-gliadins, accounting for 12% of total flour protein (Figure 1, 2B, Tables 1, 2, 5, Additional file 1). The spots, labelled in green, resolved at higher apparent molecular weights and more acidic pIs than predicted from their sequences, and were interspersed among the alpha-gliadins in the center of the gel. These gamma-gliadins represent the *Gli-A1*, *-B1 *and *-D1 *alleles that are linked to the LMW-GS *Glu-A3f*, *-B3h*, *-D3a *loci, although the specific gamma-gliadin types associated with those loci have not been described. Five spots contained more than one gamma-gliadin type and 12 spots contained one or more additional protein types, such as traces of an alpha-gliadin or LMW-GS (Additional file 1). Scaffold assigned peptides to 13 gamma-gliadin sequences, including two from NCBI nr, one from a large contig assembly and ten from Butte 86 contigs (Table 2). After manual analysis, all peptides were found to match partial (2) or complete (7) gamma-gliadin contigs assembled for Butte 86 [12] (Table 2, Additional file 1). Only one of the Butte 86 contigs was a perfect match to a gamma-gliadin sequence from the NCBI nr database. Amino acid sequence coverage of 14% to 63% was obtained for the predominant gamma-gliadins.

The most abundant gamma-gliadin type, gamma-gliadin Bu-5, was represented by four adjacent spots (134, 320, 323, 324) in the top left of the gliadin cluster (Figure 2B, Table 5). They accounted for 3.3% of total spot volume and 27.1% of the gamma-gliadins. The next most abundant was gamma-gliadin Bu-4, represented by three adjacent spots (166, 169, 337) near the bottom right of the gliadin cluster, with a total spot volume of 2.7%, representing 21.9% of the gamma-gliadins. Gamma-gliadin Bu-4 is noteworthy for having nine Cys residues, and thus is candidate for being a chain-terminating subunit of the glutenin polymer. Gamma-gliadins Bu-3, Bu-8 and Bu-10 also have nine Cys residues, but these proteins were not the predominant gliadins identified in any single spot. Gamma-gliadin Bu-2 in spot 160 was also an abundant protein, accounting for 2.2% of total protein. Gamma-gliadin type Bu-6 was identified in three adjacent spots (335, 346, 347) at the bottom right of the gliadin cluster and represented 1.9% of total flour protein. Gamma-gliadin Bu-6 is highly similar to the sequences of a pair of gliadins detected by antibodies from schizophrenic patients [64]. Lesser amounts of protein were found for other gamma-gliadin types. Gamma-gliadin Bu-1 was represented by two spots of the same relative mobility in the upper middle left of the cluster (325, 326). Gamma-gliadin Bu-11 was represented by two spots (163, 527) of similar mobility but widely spaced in apparent pI at the lower left of the gliadin cluster, and gamma-gliadin Bu-7 by only one spot (339) in the center of the bottom row of the gliadin cluster.

### Omega-gliadins

Omega-gliadins were the predominant proteins detected in 12 spots. Ten spots contained only omega-gliadins and two spots contained omega-gliadins plus another protein type (Figure 1, Table 6, Additional file 1). Three spots (69, 73, 476) were identified as omega-gliadin in other studies [40] but no peptides were detected in this analysis. These 15 spots accounted for 10% of total flour protein. The omega-gliadins were resolved between the 55,400 and 66,300 Dalton markers between pI 5.0 to 6.6, at molecular weights that were higher and pIs more acidic than predicted from their sequences. Amino acid sequence coverage of 8% to 46% was obtained. Like the gamma-gliadins, these omega-gliadins should represent the *Gli-A3*, *-B3 *and *-D3 *alleles that correspond to the LMW-GS *Glu-A3f*, *-B3h*, *-D3a *loci.

#### Omega-gliadin *Gli-B3* locus

A group of six proteins (60, 63, 69, 71, 73, 74) located between pIs 5.5 and 6.6 near the 66,300 Dalton mark represent the omega-gliadins from the *Gli-B3 *locus. They accounted for 5.1% of total flour protein. The predominant proteins in four spots (60, 63, 71, 74) were matched to TC11_28852 which encodes a ω-5 type protein. No peptides were obtained for spots 69 or 73. These omega-gliadins are referred to as the ω-5 type based on mobility in acid-PAGE gels, and have N-termini beginning SRLL. They are exceptionally rich in Gln and Pro, have many repeats of the sequence FPQQQ, and have proven to be difficult to clone. Few sequences are available in the NCBI nr or contig assembly databases. No suitable ESTs from Butte 86 were identified. However, in a previous study amino acid sequencing of thermolytic peptides from purified Butte 86 proteins revealed at least three distinct omega-gliadin proteins of this type that were resolved by 2-DE in the same location [40]. Thus there are likely to be at least three distinct proteins in these spots.

#### Omega-gliadin *Gli-A3* and *Gli-D3* loci

Omega-gliadins with predicted N-terminal sequences beginning ARQL (*Gli-A3*) or AREL (*Gli-D3*) are located on chromosomes 1A and 1D [5]. N-terminal sequences also begin KEL indicating post-translational removal of the first eight amino acids [40]. In this study, two N-terminal peptides were detected, beginning ARQ and KEL (Additional file 1). Peptides from spot 477 matched omega-gliadin Bu-D1 and Bu-D2, which are partial contigs that may represent the *Gli-D3a *loci. No omega-gliadin peptide was detected for spot 476, although 2-DE of purified omega-gliadins indicated that this spot should also contain a chromosome 1D-type omega-gliadin (Dupont et al 2000). A pair of spots (135, 391) matched omega-gliadin Bu-D5, a partial contig, and the EST [GenBank:CA714421] which may represent the *Gli-A3f *locus. Four spots (107, 113, 115, 116) were identified as omega-gliadin Bu-D2, another partial contig, which matched the complete contig TC262770. TC262770 encodes the sequence for an omega-gliadin with a single Cys that has been shown to be incorporated into the glutenin polymer. These spots were resolved just below the 55,400 Dalton marker at the acidic side of the gel. Peptides from spot 130, in the upper left of the center gliadin cluster, matched a wheat sequence for a protein [GenBank:ACN96903] that most closely resembled an omega-secalin from rye. This protein was similar but not identical to the other omega-gliadin types.

### Alpha-gliadins

Thirty-three spots contained alpha-gliadins. Alpha-gliadins were the predominant proteins in 22 of these, shown in red (Figures 1, 2C), and accounted for 20.4% of total flour protein (Tables 2, 7, Additional file 1). The alpha-gliadins were resolved as a tight cluster between apparent molecular weights of 36,500 to 50,000 Daltons and pIs of 5.7 to 7.1, all at higher molecular weights and many at more acidic pIs than predicted from their sequences. Most spots contained more than one alpha-gliadin type and many contained traces of gamma-gliadins or LMW-GSs (Additional file 1) illustrating the difficulty of cleanly separating proteins in this crowded region of the gel. A total of 34 different alpha-gliadin sequences were identified by Scaffold, with eight from NCBI nr, 16 from large EST databases, and ten from Butte 86 ESTs or contigs (Table 2). After manual analysis and interpretation of the data, the peptides were assigned to 23 proteins, 16 of which were encoded by Butte 86 ESTs or contigs. Only nine of the 689 peptides in the dataset could not be assigned to proteins encoded by Butte 86 contigs or ESTs. These were found in five spots: 177, (LQPQLPYSQPQP), 334 (LQPQHPSQQQPQEQVP, LQPQHPSQQQPQEQVPL, VRVPVPQLQPQHPSQQQPQEQVPL), 337 (HQQQQQQQQQQQQQQQPL, IILHQQQQQQQQQQQQQQQP), 342 (IILHQQQQQQQQQQQQQPLSQ, HQQQQQQQQQQQQQPL) and 467 (LQLQPFPQPQLSY) (Additional files 1, 4, 5, 6, 7, 8, 9).

Assignment of Butte 86 alpha-gliadin sequences to individual spots presented considerable challenges because of the complexity of this group of genes and proteins. One problem was that individual spots contained peptides matching up to five different alpha-gliadin sequences. Only three spots (124, 206, 344) contained a single alpha-gliadin (Additional file 1). Eight spots contained two alpha-gliadins (183, 190, 328, 329, 331, 338, 341, 420). The majority of peptides in spots 183 and 190 could be assigned to alpha-gliadin Bu-5 and alpha-gliadin Bu-14, respectively, although several peptides found in each spot corresponded to a protein encoded by a contig that was not previously described, alpha-gliadin Bu-27 (Additional files 1, 3, 5). Both are major spots that were well separated from the bulk of the alpha-gliadins (Figure 2C, Table 7). Spots 328 and 329 are abundant spots that are somewhat overlapping and the two alpha-gliadins identified in each spot, alpha-gliadin Bu-12 and alpha-gliadin Bu-17, are closely related in sequence, although the sequence of alpha-gliadin Bu-17 is incomplete (Additional file 1). Spot 341 is a major spot with peptides that corresponded to alpha-gliadin Bu-23 and alpha-gliadin Bu-8, proteins that differ by only four amino acids (Additional file 1). Other spots were very complex, containing peptides corresponding to three (342, 387, 468, 524, 546, 550), four (177, 327, 467, 525) or more (330) Butte 86 alpha-gliadin sequences. However, in most cases, the majority of the peptides could be assigned to a single Butte 86 protein. For example, 35 of the 49 peptides identified in the major spot 468 could be assigned to alpha-gliadin Bu-3 and four of these peptides were found only in this sequence (Additional file 1, 8). MS/MS coverage of the predominant protein in the 22 alpha-gliadin spots ranged from 15% to 80% with an average coverage of 54% (Table 2). In contrast to some of the other protein groups, there was little evidence for charge trains among the alpha-gliadins.

Another problem in assigning spots to specific alpha-gliadin sequences was that the Scaffold program preferentially selected sequences from the database that did not include a signal peptide instead of similar or identical sequences from Butte 86 that included the signal peptides. For example, Scaffold identified spot 190 as [GenBank:BAA12318] rather than alpha-gliadin Bu-14, an identical sequence with the signal peptide (Additional file 5). In spot 524, Scaffold selected [GenBank:CAB76964] that does not contain a signal peptide. With the exception of one additional glutamine residue not covered by any of the MS/MS peptides, [GenBank:CAB76964] is the same as alpha-gliadin Bu-3 (Additional file 9).

Incomplete contig sequences from Butte 86 also created problems with alpha-gliadin identification. Five spots (328, 329, 387, 524, 525) were identified as alpha-gliadin Bu-12 (Table 7, Additional file 1). However, two spots (524, 525) differ in both apparent size and pI from the other three spots (328, 329, 387) (Figure 2C). MS/MS data from spots 328 and 329 included additional peptides from a closely related partial sequence, alpha-gliadin Bu-17 while that from spot 387 contained peptides from a Butte 86 EST that was very similar to alpha-gliadin Bu-12, [GenBank:BQ807194]. The data indicate that there are several different alpha-gliadin Bu-12-like proteins whose coding sequences were not fully characterized in our earlier analysis of Butte 86 ESTs [13].

The most abundant alpha gliadin, spot 341, encompassed 3.3% of total flour protein (Table 7). Peptides from this spot matched alpha-gliadin Bu-23 and alpha-gliadin Bu-8, two alpha-gliadins that do not contain the major T-cell stimulatory epitopes or toxic sequences associated with celiac disease (Table 7, Additional file 1). Several other spots also contained alpha-gliadins that do not contain these celiac peptides. Five spots (328, 329, 387, 524, 525) identified as alpha-gliadin Bu-12, together comprised 3.0% of the spot volume. Spot 420, comprising 0.34% of the spot volume, was identified as alpha-gliadin Bu-27, another protein without these celiac epitopes. Altogether, these proteins encompass 6.7% of the total flour protein and 32.8% of the alpha-gliadin fraction. Most of these alpha-gliadins are encoded by genes located on chromosome 6B of hexaploid wheat (Table 7).

By comparison, the primary alpha-gliadins in 13 spots (183, 190, 206, 327, 330, 331, 338, 342, 344, 467, 468, 546, 550) contain at least some of the glia-α-9, glia-α-2, glia-α-20 and glia-α T-cell stimulatory epitopes important in celiac disease (Table 7). Together, these spots accounted for 13.1% of total flour protein and 64.2% of the alpha-gliadins. Most of these proteins are encoded by genes on chromosome 6A and 6D (Table 3). The 33-mer immunodominant peptide is found only in alpha-gliadins Bu-1 and Bu-3 (spots 342, 468), accounting for 2.5% of the total flour protein and 12.3% of the alpha-gliadins. P31-43, a peptide shown to activate the innate immune system in celiac disease, is found in alpha-gliadin Bu-5 and alpha-gliadin Bu-14, the predominant proteins in six spots (183, 190, 206, 331, 344, 546) accounting for 2.5% of the total flour protein and 12.3% of the alpha-gliadins. The p31-43 immunogenic peptide was also detected in spot 334, but this spot was not included in the totals because it also contains several gamma gliadins. The alpha-gliadin in spot 334 corresponds to a Butte 86 EST that encodes a protein that is very similar to alpha-gliadin Bu-5 and alpha-gliadin Bu-14 but is missing the N-terminus (Additional file 7). Alpha-gliadin Bu-5 and alpha-gliadin Bu-14 are encoded by genes on chromosome 6A (Table 7).

Alpha-gliadin Bu-2 encodes a protein that contains seven cysteine residues instead of the usual six, suggesting that this protein may be incorporated into the glutenin polymer. Two spots (330, 338) that differ in MW and pI contained alpha-gliadin Bu-2 and accounted for 3.2% of total flour protein and 15.7% of total alpha-gliadins (Table 7). An additional protein that is very similar to alpha-gliadin Bu-2 was also detected in a spot that contained predominantly gamma-gliadins (337).

Surprisingly few alpha-gliadin spots were identified in previous MS/MS analyses that relied only on tryptic digests. Using three proteases and improved search strategies, it was possible to detect the unique sequences that distinguished the individual alpha-gliadins. For example, spot 330 was previously identified as a gamma-gliadin based on one tryptic peptide [61] but was correctly identified in this paper as an alpha-gliadin on the basis of 26 thermolytic and 1 chymotryptic peptides.

### Farinins

Peptides from nine spots matched sequences for proteins previously termed "avenin-like-b" that were assembled from Butte 86 ESTs and named farinin Bu-1, Bu-2, and Bu-3 (Figure 1, Tables 1, 2, 8, Additional files 1, 2). Farinin was the predominant protein in eight spots (Table 8). Farinins were identified in a row of spots, Bu-1 (196), Bu-2 (193, 207, 549) and Bu-3 (336, 345), of similar mobility but differing pI below the basic half of the gliadin cluster. Farinin Bu-1 was also identified in spots 385 and 386 in the lower right of the gel. All peptides in spots 385 and 386 were derived from the C-terminal portion of the protein, suggesting that these represent a C-terminal cleavage product of farinin Bu-1 (D.D. Kasarda, personal communication). Amino acid sequence coverage was 13% to 45%. Together the farinin spots accounted for 0.9% of total flour protein (Table 2). Like the gluten proteins, the farinins were resolved at higher molecular weights than predicted.

### Purinins

Peptides from six spots matched sequences for proteins previously termed low molecular weight gliadins that were assembled from Butte 86 ESTs and named purinin Bu-1, Bu-2 and Bu-3 (Figure 1, Tables 1, 2, 8, Additional files 1, 2). The six spots, Bu-1 (542, 543), Bu-2 (219, 223) and Bu-3 (220, 227), were resolved in a group below the gliadin cluster at higher molecular weights than predicted from their sequences. Amino acid sequence coverage of 19 to 38% was obtained for the purinins, and together they accounted for 0.8% of total flour protein (Table 2).

### Triticins

Triticins were the predominant proteins in seven spots that accounted for 1.5% of total flour protein (Figure 1, Tables 2, 8). The proteins in four spots (136, 143, 348, 423) of approximately 40,000 to 55,000 Daltons at the upper right of the gliadin cluster represent the large N-terminal subunit and those in three spots (249, 253, 463) of approximately 22,000 Daltons and pIs >8.5 represent the smaller C-terminal subunit of the triticin protein. There are reported to be two genes for the complete triticin sequence [47] but post-translational modifications and/or deamidation may have caused the appearance of five spots instead of two for the large subunit. Peptides from three of the large subunit spots (136, 348, 423) were matched to a single contig TC11_285558, and peptides from spot 143 were matched to different contig, TC11_264477 as well as to the EST sequence [GenBank:DR736644]. Peptides from the small subunit spots were matched to TC11_264477 and TC11_285558. There were insufficient ESTs from Butte 86 to assemble reliable contigs, although Butte 86 ESTs matched portions of the sequences above. Amino acid sequence coverages of 17 to 36% were obtained (Table 8).

### Globulins

Only a small proportion of flour proteins are globulins, and they are more easily studied in globulin-enriched saline extracts of flour. However, 11 globulin spots were sufficiently abundant to be detected and quantified in this study of endosperm-derived flour (Figure 1, Tables 1, 2, 8, Additional files 1, 2). Peptides were assigned to eight globulin sequences, including three Butte 86 contigs. Globulin-1 (*Glo-2*) was the predominant protein in one spot (218), globulin-2 in five spots (99, 103, 104, 106, 121) and proteins termed embryo globulins or products of the *Glo-3 *locus in three spots (180, 184, 309). Together, they comprised only 0.4% of total flour protein (Table 2). Amino acid sequence coverages of 10 to 48% were obtained.

Peptides from spot 218 corresponded to a globulin sequence [GenBank:ABG68030] and peptides from the mixed spot 530 matched a similar sequence [GenBank:AAM77589] termed alpha-globulin or seed globulin (Figure 1, Table 8). These spots were resolved below the LMW-GS, towards the basic side of the gel at a somewhat greater molecular weight than predicted from their sequences (Figure 1).

Peptides from five spots matched three contigs encoding globulin-2 proteins for which there are at least partial Butte 86 sequences [51] (Additional file 2). Two spots (103, 106) corresponded to contig Bu-18428, spot 99 to contig Bu-17366 and two spots (104, 121) to contig Bu-17295. The globulin-2 types have substantial similarity to known allergens from other plants [51]. These spots were resolved near their predicted molecular weights of 54,000 Daltons between pIs 5.9 and 7.0, above the LMW-GS cluster.

Peptides from three spots (180, 184, 309) corresponded to embryo globulin sequences that closely resemble the *Glo-3 *[52] alleles. Scaffold assigned the peptides in spot 180 to four different embryo globulin contigs, those in spot 184 to two contigs and those in 309 to a single contig (Additional file 1). Spots 180 and 184 were found to the lower right of the LMW-GS and spot 309 was found to the upper right of the LMW-GS in Figure 1.

### Grain-softness proteins and puroindolines

Two spots (248, 271) with similar basic pIs but different molecular weights were identified as puroindoline b (Figure 1, Tables 1, 2, 8, Additional file 1). Two additional spots (174, 275) in the same region of the gel were identified as grain-softness proteins. Together these accounted for 0.3% of total spot volume (Table 2). Spots 271 and 275 resolved near their predicted molecular weights of 14,800 and 16,400 Daltons, but spots 174 and 248 resolved at considerably higher molecular weights than predicted.

### Alpha-amylase/protease inhibitors

Twenty spots contained alpha-amylase/protease inhibitors as the predominant protein, accounting for 4.1% of the total flour protein (Figure 1, Tables 1, 2, 9, Additional file 1). All spots were in the lower left to lower center region of the gel at the predicted molecular weights shown in Table 9. Although the alpha-amylase/protease inhibitors were well separated from other protein types, there was considerable overlap among members within the group. Peptides were matched to a total of 16 different sequences by Scaffold, with nine from NCBI nr and seven for contigs from EST assembly databases (Table 9). MS/MS coverages for the predominant alpha-amylase/protease inhibitors in each spot ranged from 20 to 90% with an average coverage of 58.2%.

#### WMAI

The monomeric alpha-amylase inhibitor, WMAI, was found in 2 spots (289, 528) that differed significantly in pI (Figure 1, Table 9). The spots accounted for 0.5% of total flour protein. Scaffold matched all peptides from both spots to the same protein sequence [PRF:223520].

#### WDAI

Seven spots contained dimeric alpha-amylase inhibitors, referred to as WDAI (Additional file 1). Three protein sequences were represented, two from NCBI and one from a large contig database. A 0.19 type inhibitor, [SwissProt:P01085], was the predominant protein identified in spot 312, but was also a significant component of spots 280, 283, 285 and 313, all of which had similar molecular weights but different pIs (Figure 1, Table 9, Additional file 1). The 0.53 type inhibitors, TC11_338524 and [GenBank:AAV91972], were major components of spots 283 and 286, respectively. TC11_338524 was also identified as a minor component of spot 281, and [GenBank:AAV91972] was also identified in spot 283. The dimeric alpha-amylase inhibitor spots accounted for 1.0% of total flour protein.

#### WTAI

Tetrameric alpha-amylase inhibitors, often referred to as CM types, were the predominant protein in ten spots that were clustered in several regions of the gel. The WTAI-CM3 type proteins were found in three spots (264, 265, 272) and corresponded to two protein sequences, [SwissProt:P17314] and RS_UWI_15430 (Table 9, Additional file 1). The other WTAI subunits were represented by single protein sequences. The WTAI-CM16 and WTAI-CM17 subunits were clustered in four spots at the acidic end of the gel that differed in both MW and pI (266, 274, 282, 284) while WTAI-CM1 and WTAI-CM2 were the predominant proteins in three spots (280, 285, 313) that overlapped with some of the WDAI spots. The WTAI spots accounted for 1.7% of total flour protein.

#### WASI, CMx and WCI

An inhibitor of endogenous alpha-amylase, WASI, was found in a single spot (244) above the 21,500 Dalton mark and corresponded to [SwissProt:P16347] (Figure 1, Table 9). Three different forms of the CMx type protease inhibitors also were found, corresponding to three protein sequences from contig databases. TC11_308146 was a major component of spot 290 and a minor component of spots 281 and 286 and TC11_309398 was the predominant protein identified in spot 281, but was also found in spots 286 and 290 (Figure 1, Table 9, Additional file 1). These spots had similar molecular weights but different pIs. TC11_320696 was a minor component of two spots (280, 313) that contained WTAI-CM1 and WTAI-CM2 and the WDAI [SwissProt:P01085]. Two protein spots (277, 278) also corresponded to a putative chymotrypsin inhibitor, WCI [GenBank:CAD19440], although one of these also included several unrelated proteins. A protein that is weakly similar to a putative trypsin inhibitor from *Triticum monococcum *was also identified as a minor component of two spots (278, 286) (Additional file 1).

Many of the alpha-amylase/protease inhibitors were detected in multiple spots that differed in pI but not in apparent molecular weight. In contrast, WTAI-CM16 and WTAI-CM17 were each detected in spots that differed in apparent molecular weight. It is possible that the proteins with the greater apparent molecular weights (spots 266, 274) were glycosylated forms of WTAI-CM16 and WTAI-CM17. A glycosylated form of WTAI-CM16 was reported previously in durum wheat and was found to be considerably more reactive with IgE from patients with baker's asthma than the non-glycosylated protein [65]. It is notable that the spots with the lesser MWs were at least ten-fold more abundant in the flour (Table 9).

### Serpins

Fourteen spots (146, 147, 148, 149, 150, 151, 152, 154, 158, 159, 162, 397, 398, 399) were identified as serpins accounting for 1.6% of total flour protein (Figure 1, Tables 1, 2, 9, Additional files 1, 3). These spots are clustered to the left of the gliadins, at close to their predicted molecular weights of 37,000 to 43,000 Daltons and pIs of 5.1 to 5.4. There were not sufficient Butte 86 ESTs to assemble contigs encoding complete serpin proteins. However, similarities between sequences selected by Scaffold and Butte 86 sequences are noted. The Butte 86 sequences were not included in the original databases used for MS/MS analysis. Five serpin categories have been described. Serpin type 1a was represented by peptides from three spots (159, 398, 399), with peptides from spot 159 assigned to three sequences including serpin Bu-4 and Bu-5, and peptides from spots 398 and 399 assigned to serpin Bu-3, which is similar to [SwissProt:P93693]. Serpin type 1b was identified in six spots (146, 147, 148, 155, 158, 162) and corresponded to serpin Bu-1, similar to [GenBank:ACN59483]. For spots 162 and 158 it was not possible to distinguish between serpin Bu-1 and Bu-4. Serpin type 2b was represented by two spots (151, 154) matching serpin Bu-5, similar to [GenBank:CAA72274]. Serpin type 1c was represented by spots 149 and 397, matching serpin Bu-2, similar to [SwissProt:Q9ST58]. Serpin type 3 was represented by spots 150 and 152 matching serpin Bu-7, similar to [GenBank:ACN59484]. Amino acid sequence coverages of 18 to 70% were obtained (Table 9).

### Other inhibitors

Two other inhibitors were identified (Figure 1, Tables 1, 2, 9, Additional file 1). Peptides from spot 201 at the far right of the gel corresponded to three different sequences for tritin, a ribosomal inhibitor, with 0.15% of total spot volume and amino acid coverages of 52% to 64% for the three sequences. This spot was resolved above the predicted molecular weight of 30,000 Daltons. Peptides from spot 205 corresponded to the xylanase inhibitor XIP-1 [PDB:1OM0] with 0.1% of total spot volume and amino acid coverage of 54%. This spot was resolved near its predicted pI of 8.3 and somewhat above its predicted molecular weight of 30,000 Daltons.

### Enzymes

Seven spots (64, 92, 93, 94, 462, 476, 537) were identified as beta-amylase and minor amounts of beta-amylase were detected in other spots (108, 110, 299, 436) (Figure 1, Tables 1, 2, 10, Additional files 1, 2). All of these spots were at the left of the gel between the 55,400 and 66,300 Dalton markers. These proteins represent the products of the three alleles β-*Amy-A1*, β-*Amy-B1*, β-*Amy-D1*. Although Butte 86 contigs for three beta-amylase sequences were assembled (beta-amylase Bu-1, Bu-2 and Bu-3) and proteins corresponding to all three contigs were identified, it is not known which sequence corresponds to which allele. Peptides from two spots (476, 537) corresponded to beta-amylase Bu-1, those from two spots (94, 462) to beta-amylase Bu-2, and those from three spots (64, 92, 93) to beta-amylase Bu-3, with some cross-contamination between the spots. The largest spot, spot 476 is likely to consist mainly of the Bu omega-gliadin D-1 protein, but traces of beta-amylase were easier to detect by MS/MS than major amounts of an omega-gliadin. Excluding spot 476, the beta-amylase spots contributed 0.5% of total spot volume. Amino acid sequence coverages ranged from 13 to 60% (Table 10).

Eighteen other enzymes were identified (Figure 1, Tables 1, 2, 10, Additional file 1). Unlike the gluten proteins, most enzyme spots corresponded to their predicted molecular weights and pIs. Two spots (89, 128) were identified as ADP-glucose pyrophosphorylase large subunit and two spots (108, 110) as ADP-glucose pyrophosphorylase small subunit. Two spots (232, 455) contained peptides from chitinase and two spots (175, 176) contained malate dehydrogenase. Two spots (239, 479) contained peptides from the so-called 27 K protein that is similar in sequence to a thiol reductase. Single spots were identified as alanine aminotransferase (118), mitochondrial ATP-synthase β-subunit (109), dehydroascorbate reductase (241), glucose/ribitol dehydrogenase (202), glyoxalase (lactoylglutathione lyase) (178), ketol acid reducto isomerase (436), methionine synthase (450), protein disulfide isomerase 3 (299), pyruvate orthophosphate dikinase (371), sucrose synthase (475), thiamine biosynthetic enzyme (189), and triose phosphate isomerase (225). Unlike most of the enzymes, the spot corresponding to pyruvate orthophosphate dikinase (371) was resolved at a higher molecular weight than predicted, suggesting that the protein may be modified post-translationally. In all, these accounted for 1.4% of total flour protein. In addition, nine other enzymes were minor components of other spots (Table 10, Additional file 1). Most are likely to be the trace remains of enzymes involved in starch and protein biosynthesis during the later stages of grain fill, along with enzymes involved in the response to attack by pathogens. It is unknown to what extent they retain activity. Amino acid sequence coverages of 10 to 83% were obtained.

### Other proteins

The remaining 6 minor proteins include elongation factor EF1A (311), initiation factor Eif4A (413) and HSP70 (533), which are involved in RNA and protein synthesis, and LTP Bu-2 (295). A thaumatin-like protein was identified in spot 456 along with chitinase. These spots accounted for 0.4% of total flour protein. Amino acid sequence coverages of 14 to 39% were obtained (Table 10).

## Discussion

Improved ability to identify and distinguish among flour protein types is essential for understanding genetic and environmental effects on functional properties of flour as well as to clarify the role of specific proteins in celiac disease and food allergies. However, the utility of one of the major proteomics techniques, MS/MS, is limited for studies of flour proteins unless the following three conditions can be met: proteins must be cleaved into fragments suitable for MS/MS analysis, the sequence database must be adequate to discriminate among highly similar proteins, and the data analysis must account for the presence of highly similar proteins. In this paper, a three protease approach was used to improve protein cleavage, MS techniques were adjusted for the use of non-tryptic peptides, several strategies were used to improve the sequence database interrogated with the MS spectral data, and improved strategies were used for data analysis.

Living organisms have large dynamic ranges for protein abundance and small quantities of a particular enzyme or regulatory protein may play important roles. However, white flour is mainly the product of a dead storage tissue, and the focus of this study was to identify and quantify the abundant proteins that contribute to the functional properties of wheat flour in doughs and baked goods. Ironically it has been far easier to identify proteins of much lesser abundance in water and salt-soluble subfractions of wheat flour or endosperm than to identify the abundant alcohol-soluble gluten proteins by 2DE MS/MS [23,25,66,67].

### Determination of the number of expressed genes

The wheat genome is gradually being sequenced and the complex homoeoallelic loci are being dissected and described. The evolution and structure of the HMW-GS loci have been elucidated in some detail [33] and recent papers outline the constituents of the LMW-GS alleles [35,68,69]. Gene duplications apparently occurred before and after the origins of the three bread wheat genomes, leading to multiple homoeologs at complex loci that include expressed and unexpressed genes and pseudogenes. Precise determination of actual protein composition is an essential step in elucidating the patterns of gene expression for the storage proteins and complements efforts to sequence the expressed genome. In this paper, we identified five HMW-GS, 22 LMW-GS, 13 gamma-gliadins, seven omega-gliadins and 23 alpha-gliadins. Previously through EST analysis, we identified coding sequences for twelve full-length and seven partial alpha-gliadins as well as nine full-length and two partial gamma-gliadin sequences expressed in Butte 86 [12,13]. We also identified five distinct serpin proteins, in agreement with a previous study [55]. In agreement with other reports, there appeared to be only 2 expressed copies of the triticin gene [47]. We identified three each for beta-amylase and purinin, and detected three for farinin. We identified 16 members of the alpha-amylase/protease inhibitor family.

### Quantification of wheat flour proteins

Quantification of flour proteins is often based on methods that utilize solvent-based sequential fractionation and quantification of the subfractions, and may include additional separation of the subfractions by methods such as RP-HPLC and 2-DE [61]. Such methods separate proteins by type, but rarely by individual protein. In contrast, for this paper a one-step extract was separated and quantified by 2-DE. The 233 proteins identified in this study account for 93% of the total protein in milled white flour from the US cultivar Butte 86. Gluten protein comprised 78.2% of the flour protein with gliadins contributing 43.1% and glutenin subunits contributing 35.1%. Alpha-gliadins were the most abundant protein type, accounting for 20.4% of total protein and 47.3% of the gliadin fraction. LMW-GS and HMW-GS were of similar abundance, comprising 17.9 and 17.1% of the flour protein, respectively. Because the study associated specific gene sequences from Butte 86 with individual protein spots, the proportions of gliadins containing an odd number of Cys residues could be estimated. Gliadins that might serve as chain terminating glutenin-subunits comprised 7.2% of the total flour protein in Butte 86. The data also revealed that the A genome makes a significantly smaller contribution than the B and D genomes to the final amounts of HMW-GS, LMW-GS, alpha-gliadins and omega-gliadins in Butte 86 flour. Proteins encoded by the A genome comprised only 14.0% of the total HMW-GS, 12.2% of the LMW-GS, 12.1% of the alpha-gliadins, and 10.2% of omega-gliadins. Other storage proteins, including the triticins, farinins, purinins and globulins, together comprised about 3.7% of the total flour protein. Grain softness proteins and puroindolines comprised only 0.3% of total flour protein, despite their importance in determining kernel hardness. The alpha-amylase/protease inhibitors and the serpins were also complex groups of proteins contributing 4.1 and 1.6% of total flour protein, respectively. Twenty-six enzymes, inhibitors and other proteins were also identified in this study and together comprised about 2.7% of the flour protein.

### Evaluation of number and quantity of potential allergens and proteins involved in celiac disease

Comprehensive proteomic analysis of the wheat flour proteins provides insight into the prevalence of potential allergens and proteins that might elicit symptoms of celiac disease. Certain proteins of moderate to high abundance have been implicated in wheat allergies, including proteins in the omega-gliadin, alpha-amylase inhibitor and serpin families. It should now be possible to evaluate the allergenic potential of individual proteins within these complex families. It is also interesting that a number of proteins that comprise less than 0.1% of wheat flour protein, including LTP and the 27 K protein have been classified as allergens by the International Union of Immunological Societies. Detailed knowledge about the sequences of the wheat flour proteins and their relative abundance is also of importance in better understanding celiac disease. While proteins within all of the major gluten protein families may play a role in celiac disease, the analysis of the alpha-gliadins illustrates the potential for determining the prevalence of celiac epitopes expressed in a particular wheat cultivar.

### Limitations to identification of highly similar proteins by MS/MS

Identification of proteins by MS/MS is based on matching the spectral fragmentation patterns of actual peptides to masses predicted by *in silico *digestion and fragmentation of proteins and peptides predicted from sequence databases. Even when MS/MS results appear to have a high probability of being correct, there are limitations to this method for distinguishing among highly similar proteins that are the product of homoeologous genes, because of the manner in which existing data analysis programs handle the data. Since the two programs for spectral analysis, XTandem! and Mascot did not give identical results [59,70] both were utilized and the results integrated using Scaffold. However, there were considerable challenges when protease digests of two closely related proteins shared many identical peptides. Using spot 125 as an example, peptides from two distinct LMW-GS were detected and matched to the sequences [GenBank:AAB48469] and [GenBank:BAD12055]. A total of 21 distinct peptides were detected, based on 22 spectra (Additional file 1). Of these, 12 peptides were found only in [GenBank:AAB48469], five peptides were found only in [GenBank:BAD12055] and the remaining four peptides were found in both sequences. Scaffold calculated that there were 16 "unique" peptides from [GenBank:AAB48469], rather than 12 unique peptides and four shared peptides, because Scaffold first assigned all possible peptides to the protein with the greatest number of peptides and used the term "unique" to describe all 16 peptides. The remaining proteins that could not be assigned to the first protein were then assigned to the second protein. Since it is not possible to determine which of the shared sequences came from which protein, the number of peptides from the dominant sequence may be overestimated and the number from the minor sequence(s) underestimated.

It is obvious that if samples contain two or more similar proteins, MS/MS may or may not detect the few peptides that distinguish them. It is essential to obtain as many peptides as possible from each protein to maximize sequence coverage. In a recent MS/MS study, the number of peptides identified for the wheat storage proteins was greatly increased by the use of the three enzymes, chymotrypsin, thermolysin and trypsin, rather than any single protease [59]. It also is essential that the database have as much DNA or protein sequence data as possible in order to recognize the distinguishing peptides.

While Scaffold determined probabilities of 100% for most identifications, it is likely that the usual rules do not apply to protein groups as complex as the gluten proteins. We accept that there was 100% probability that the correct protein types were identified, but not that the exact sequences were correct. The present study confirmed our previous observations that it was better to perform a separate MUDIT analysis of each 2-DE spot than to analyze the combined peptide data [59] because programs such as Scaffold attempt to determine the minimum number of proteins that can be accounted for by the entire data set and will combine data across spots.

### Inconsistent nomenclature

Another limitation encountered in this study was that the computer-assigned name for each protein was, reasonably, the name associated with that sequence in the original database. Generally, these names were correct, but in a number of cases mistaken, misleading or inconsistent identities persist in the NCBI nr database, along with inconsistencies between protein name and gene locus. Also, some gene sequences were not given names, for example the genomic sequence for HMW-GS Bx7 was reported in NCBI as "unknown". It was essential to verify all the identities of database-assigned names.

### Value of cultivar-specific databases

Rapid evolution of the gluten proteins has led to minor differences in amino acid sequence that made it surprisingly difficult to use existing sequence databases for precise identification of gliadins and LMW-GS [10,12,13,31]. Initially, our MS/MS data were used to interrogate a database that included the NCBI non-redundant database and extensive contig databases constructed from thousands of ESTs obtained from multiple wheat varieties, but many large spots were not identified at all or not identified precisely despite using that extensive database. Addition of sequences specific to Butte 86 greatly improved the results, both in assigning a general protein type to the spot, and in improving sequence coverage sufficiently to distinguish among similar homeologous proteins. One might think that an alpha-gliadin spot would at least be identified as an alpha-gliadin by MS/MS, even if exact sequences were not in the database. However, the programs that match spectra to sequence data require close matches. Small differences, such as a few extra Gln in the repetitive region of a gliadin sequence or one amino acid change that altered a proteolytic site result in proteolytic or fragmentation patterns that are not recognized [12,13]. In this paper, extensive sequence coverage was realized for the Butte 86 sequences and they were often clearly distinguished from similar homologs in the NCBInr database. Of the 168 sequences selected by Scaffold in this study, 34% were from NCBI nr, 38% from large contig databases and 29% from Butte 86. However, nearly 40% of the sequences selected after manual analysis of the data were from Butte 86.

In order to estimate protein sizes and pIs, it was necessary to manually edit the contig sequences. Since most wheat flour proteins are secreted into the ER, it was also necessary to predict the size of the signal peptide and recalculate molecular weight and pI based on the sequence of the mature protein.

### Charge trains, mobility and pI

Charge trains are commonly observed in 2-DE of flour proteins. In this paper, potential charge chains were recognized because of the high and accurate sequence coverage obtained for most spots. For example, 40 spots represented only 5 HMW-GS genes, and seven spots appeared to represent a single LMW-GS gene. The presence of charge trains has been attributed to an artifactual modification of the sample prior to and during electrophoresis [71]. It has been pointed out, however, that with normal sample handling modifications such as carbamylation and deamidation do not occur [72,73]. Examination of our mass spectrometry data revealed that a number of the glutamines in the HMW-GS are deamidated. In one study, adjacent spots in a charge train differed in mass by 1 Dalton. This was proposed to result from deamidation of asparagine or glutamine residues attributed to naturally occurring *in vivo *processes [74]. As some of the HMW-GS are deamidated it is possible that the charge trains (which are highly reproducible) are the result of naturally occurring *in vivo *modifications. There was not sufficient coverage in all of the HMW-GS to allow us to determine whether this was true. Other sources of charge train formation could be the loss of DTT during IEF, resulting in oxidation of methionines [75] or alkylation of SH-groups by acrylamide and linkage of previously reduced SH-groups. Desulfuration (β-elimination) can also lead to a number of unexpected events and even protein degradation [71]. It has been suggested that thiol artifacts can be prevented by alkylation of the proteins prior to IEF [73]. However, a test gel of alkylated proteins did not decrease the number of HMW-GS spots. Conformational changes during electrophoresis have also been proposed to cause apparent charge trains [76].

Failure to understand that multiple spots derive from the same protein can lead to misinterpretation of the results of experiments in which 2-DE is used to compare the samples. There are several examples in the literature where it is suggested that changes in spot volume for minor spots indicate a significant effect of genetics or environment when the authors did not acknowledge that spots may be members of charge trains [28,77,78]. Also, the occurrence of charge trains increases the difficulty in deciding if two spots represent the product of a single gene or two highly similar homeologs, since there is rarely 100% peptide coverage and all possible sequences for these complex gene families are not known.

Another unusual feature of the gliadins and glutenin-subunits is their tendency to be resolved at higher molecular weights than expected based on their sequences and known masses, which may be explained by failure to bind as much SDS as a typical protein and/or failure to assume a compact rod shape during electrophoresis [6,40]. The results in this paper also indicated that most gluten proteins were resolved at more acidic pIs than expected.

### Evidence for protein processing

Generally there seems to be little post-translational processing or even degradation of the flour proteins, other than cleavage of the signal peptide from the secreted proteins. There is little glycosylation of the endosperm proteins, although this may account for small variations in size for a few of the spots, such as WTAI-CM-16 and WTAI-CM-17. Evidence for only one protease was observed, an aspartic protease that was a minor component of six spots (312, 313, 335, 339, 345, 347) (Additional File 1). However, a few examples of processing were noted, such as the variability of the N-terminus of the LMW-GS. N-terminal sequencing of the LMW-GS determined that they begin with Met, Ser or Ile as in the METSHIP, METSCIP, SHIP, or ISQQ types [16,38]. However, the predicted N-terminal sequences for the mature proteins are QMETSHIP, QMETSCIP, QMENSHIP, QISQQ, etc. as determined by algorithms for signal peptide cleavages. N-terminal sequences beginning with M appear to result from a non-predicted cleavage after the Q, and sequences beginning SHIP would result from an asparagine protease cleavage after the N in QMENSHIP [6,79]. It is likely that sequences beginning Q were N-terminally blocked and not observed in N-terminal amino acid sequencing studies. In this paper, one N-terminal peptide beginning QISQQQ was observed, one beginning QMET, two beginning MET, and six beginning SHIP (Additional file 1), confirming that the N-termini QMET and QISQQQ exist and agreeing with previous results showing additional processing of the LMW-GS. Similarly, we found N-termini beginning QLETT and LETT for the farinins and the processed N-terminus KEL for an omega-gliadin. Other examples of post-translational processing are the cleavage of farinin Bu-1 and of triticin into two subunits of different sizes.

### Prospects for rapid analysis of wheat flour proteins

This paper illustrates a method to overcome many of the difficulties of identifying the total complement of flour proteins. However, the combination of 2-DE, three protease digestions of each spot, and iterative analysis by MS/MS is time consuming and costly. 2-DE is of great value for comparing multiple samples, and once a 2-DE map is obtained for a particular wheat cultivar the information can be used as the basis for many future experiments. Therefore, the exact methods used in this paper may be useful to researchers who rely heavily on 2-DE and have access to MS/MS. However, it is highly desirable to continue to develop easier and less expensive methods to analyze flour proteins.

DNA analysis may be the basis for the least expensive and most rapid methods for breeders to predict flour protein composition [19]. However, actual identification and quantification of proteins is necessary in order to determine precisely how environment influences protein composition and to relate the exact protein composition to baking and mixing quality. Expression levels of genes may be as important as exact sequence type in determining their influence on flour quality. Cheaper, more rapid methods for protein determination by MS may become available, but will require comprehensive sequence databases. Improved methods for more rapid analysis of protein composition by MS may include MUDPIT analyses of complex protein samples, in which a sample that contains many proteins is digested with a protease, the peptides are analyzed by MS/MS, and computer programs then used to assign the peptides to individual sequences. Another MS method utilizes MALDI analysis of a complex protein sample to predict protein composition based on the masses of the intact proteins [31]. All of these methods require an accurate and complete DNA database that includes the sequences for all flour proteins found in that wheat cultivar. We recommend that future studies of flour protein composition begin with a thorough cDNA sequencing project aimed at extensive coverage of the gliadins and glutenins for the cultivars being compared, as this can now be done fairly easily, is relatively inexpensive, and in the long run will greatly simplify analysis of the flour proteins.

## Conclusions

This is the first report in which the majority of the abundant flour proteins were identified with sufficient coverage to assign them to specific gene sequences and determine their expression levels. The ability to identify and quantify individual flour proteins makes it possible to measure the precise effects of genotype, environment, and fertilizer regimen on flour protein composition, including their effects on the subunit composition of the glutenin polymer. It also enhances the ability to determine the prevalence of epitopes important in celiac disease and potential allergens in flour. These studies provide new insight into a major food ingredient that contributes to human nutrition and human health throughout the world.

## Methods

### Plant materials

Plants of the US hard red spring wheat *Triticum aestivum *'Butte 86' were grown at 24°C days, 17°C nights as described in detail previously [60]. Briefly, the plants were grown in a climate-controlled greenhouse with 16 hr days and 8 hr nights and watered by drip irrigation with 0.6 g.l^-1 ^Plantex fertilizer (NPK, 20:20:20). Grain from three sets of pots was harvested at maturity and grain samples of 100 g per set were milled to flour with a Brabender Quadrumat Junior (South Hakensack, NJ) at the Hard Winter Wheat Quality Laboratory (US Department of Agriculture, Agricultural Research Service, Manhattan, KS) [61]. Flour was stored in sealed containers at -80°C.

### 2-DE of total flour proteins

Proteins in each of the three flour samples were separated and analyzed by 2-DE as described previously [80,81]. Proteins were extracted with SDS buffer (2% SDS, 10% glycerol, 50 mM DTT, 40 mM Tris-Cl, pH 6.8), which extracted a greater percentage of protein from flour than other methods and facilitated removal of starch. Following addition of 800 μl of SDS buffer to 50 mg of flour and incubation for 1 h at room temperature with intermittent mixing, insoluble material, largely starch, was removed by centrifugation at 16,000 *g *for 10 min (Eppendorf Centrifuge 5415 C, Brinkman Instruments, Inc., Westbury, NY). Triplicate 5 μl samples were taken from the supernates for protein analysis. Proteins were precipitated by addition of 4 vol of cold (-20°C) acetone to remove the SDS, which interferes with protein determination and prevents separation of proteins by IEF. Following incubation overnight at -20°C and centrifugation at 14,000 rpm for 10 min at room temperature, cold acetone was pipetted onto the pellets, samples centrifuged as above, and the acetone pipetted off the pellets. Pellets were vacuum dried (Speed Vac DNA 110; Savant Instruments, Inc., Farmingdale, NY). Protein in the 5 μl samples was solubilized in 0.1 N NaOH and quantified by the procedure of Lowry et al. [82] with BSA as standard. Protein to be analyzed by IEF was solubilized in urea buffer (9 M urea, 4% NP-40, 1% DTT, and 2% ampholytes) to a final concentration of 3 μg protein/μl.

Proteins in each of the three flour samples were separated and analyzed in triplicate (9 gels total) by 2-DE. The first dimension IEF capillary tube gels contained 9.2 M urea, 4% (total monomer) acrylamide: Bis, 2% Nonidet P-40, 2% 3-10 Iso-Dalt Grade Servalyts (Crescent Chemical Co., Islandia, NY), 0.015% ammonium persulfate and 0.125% TEMED. The upper electrode (anode) buffer was 0.2% (v/v) sulfuric acid and the lower electrode buffer (cathode) was 0.5% (v/v) ethanolamine. Because the anode buffer was acidic, the leads from the electrophoresis cell were reversed at the power supply. The gels were prefocused at 200 V for 10 min, 300 V for 15 min and 400 V for 15 min. Samples containing 18 μg of protein were loaded at the acidic end of the IEF gel and overlaid with 5 M urea. For protein *p*I determinations, 3 μl of 2-D SDS-PAGE Standards (BioRad) were added to the sample. IEF gels were run at 500 V for 10 min and then at 750 V for 1 h. Gels were extruded into microcentrifuge tubes using the BioRad tube gel ejector attached to a 10 ml syringe without buffer. Gels were placed in microcentrifuge tubes and equilibration buffer (2.3% SDS, 10% glycerol, 0.05% DTT, 62.5 mM Tris-Cl pH 6.8) was added. Tubes were immediately placed in dry ice and the frozen gels stored at -70°C. Proteins were separated in the second dimension by SDS gel electrophoresis (XCell SureLock Mini-Cell electrophoresis system; Invitrogen Corp., Carlsbad, CA). IEF gels were thawed, placed on top of Novex NuPAGE gels (Bis-Tris 4-12% acrylamide 1 mm thick gels with 2-D well; Invitrogen Corp.), and overlaid with 45 μl of equilibration buffer. Three μl of molecular weight markers (Mark 12 Unstained Standard; Invitrogen Corp.) were loaded into the 2-D well. The SDS gels were run with NuPAGE MES SDS running buffer (Invitrogen Corp.) for 48 min at 200 V. The 2-D gels were stained with Coomassie G-250 (Sigma, St. Louis, MO), destained in water for 2 h, and stored at 4°C in 20% ammonium sulfate [83]. The stained gels were digitized with a calibrated scanner (PowerLook III, UMAX Technologies, Inc., Dallas, TX) at 300 dpi with the same settings for all gels and protein spots matched and quantified by computer analysis (Progenesis PG240 version 2006, Nonlinear Dynamics, Newcastle upon Tyne, UK). The 2-DE analysis software aligned gels, detected spots, subtracted background, and normalized spot volumes. Background subtraction corrects for staining variations across the gel to accurately quantify protein present in a spot. Normalization of spot volume corrects for protein quantity variation among gels and is a calculation of the individual spot volume divided by the total spot multiplied by 100. The means and standard deviations for spot volumes of individual proteins were similar among the three replicates. Averages and standard deviations for the three biological replicates were then determined.

### Protein identification

Each distinct protein spot was excised from at least three 2-DE gels in order to digest them separately with trypsin, thermolysin and chymotrypsin. MS/MS analysis of enzymatic digests of protein spots was carried out with a QSTAR Pulsar *i *quadrupole time-of-flight mass spectrometer (Applied Biosystems/MDS Sciex, Toronto, Canada) that was equipped with a nano-electrospray source and nano-flow liquid chromatograph [27]. Automatic determination of the appropriate collision energy (relative to m/z) was carried out by the Analyst QS 1.1 software. When analyzing samples digested either with chymotrypsin or thermolysin the intercept of the collision energy values was decreased by eight units relative to that used with trypsin. The spectra from each digest were used to interrogate a "SuperWheat" database (Version # 100211) as described in [59]. For this study, the "SuperWheat" database was constructed by concatenating the following publicly available databases; NCBI non-redundant green plant protein sequences (download date: 2/11/2010) [84], nucleotide sequences translated in all six reading frames of contigs from TaGI Releases 10.0 and 11.0 [63], US Wheat Genome Project [85], HarvEST 1.14 (WI all NSF "stringent" assembly from 05/08/04) [86], NCBI Unigene Build #55 [84], and all ESTs from Butte 86 developing grain, as well as translated sequences (reading frame only) of 94 Butte 86 contigs (Additional file 2), including those for alpha-gliadins and gamma-gliadins [12,13]. Additionally the database contained a list of proteins known to be common laboratory contaminants [87] and sequences for thermolysin. The "SuperWheat" database contained 2,094,746 protein sequences. Two search engines, X!Tandem [57,87] and Mascot version 2.1 (Matrix Science, London, UK) [88,89] were used to match the peptide mass spectra to spectra generated *in silico *from database peptides. It has been demonstrated that Mascot and X!tandem yield somewhat different results [59,70]. Mascot identified significantly more tryptic peptides than did X!tandem, while X!tandem identified more peptides from proteins digested with thermolysin or chymotrypsin. Scaffold Version 2_02_04 [62] was used to assemble and visualize MS/MS derived peptide and protein identifications. A "subset" database was generated from the initial search of the SuperWheat database by exporting from Scaffold all protein sequences that had a 20% or greater probability of being a match. Appended to these 2,134 sequences was an equal number of decoy protein sequences from the archaeobacter *Jannaschia *sp, translated sequences from the set of Butte 86 contigs not already included in the subset database, and the set of common protein contaminants. A "second pass" search [90,91] was conducted with both search engines and the results assembled and validated with Scaffold. Identifications of proteins were required to meet the following criteria: at least two peptides having a parent mass tolerance threshold of less than or equal to 100 ppm and a greater than 90% peptide probability as specified by the Peptide Prophet algorithm [92]. Scaffold Version 3.00.03 was used to compile the final set of MS/MS based peptide and protein identifications, using the MUDPIT algorithm to independently analyze the data for each spot. The false discovery rate was generally found to be 0.0% under the filter settings used. The data associated with this manuscript may be downloaded from ProteomeCommons.org Tranche using the following hash:

hCc5INiKGH0m4DEfxLbShm1F+us+JyZ/HENjkOTlGcni8NmnyoEwU5i7Onf/Po2kNtnP10SCdgODD6Swo0hgF69d3dIAAAAAAAB6hg==

### Manual analysis of the MS/MS data

After completing the analysis using Scaffold, all data were interpreted manually. Additional files 4, 5, 6, 7, 8, 9 show the peptides found, the sequence assignments given by Scaffold, any different sequence assignment made after final interpretation of the data, and the reason for that assignment. Protein assignments displayed by Scaffold may represent mature proteins, unprocessed proteins or proteins translated from unedited contigs. Since most flour proteins are produced through secretory pathways, in almost all cases it was necessary to manually determine the sequence of the mature protein and estimate the molecular weight, pI, and percentage coverage (Tables 3, 4, 5, 6, 7, 8, 9, 10, Additional file 1). Signal peptide length and cleavage sites were estimated using the Target P program with the choices of "green plant" and 'predict cleavage" [93]. Molecular weight and pI were estimated from the edited mature sequence using the ProtParam program on the Expasy-Tools database [94]. Where the complete sequence was not available, highly similar sequences of complete proteins in the database were used to make these estimates, as indicated.

When a closely related sequence from Butte 86 accounted for all of the assigned peptides it was substituted for the sequence selected by Scaffold. Alignments between Scaffold selected sequences and protein sequences deduced from Butte 86 ESTs or contig consensus sequences are shown in Additional files 4, 5, 6, 7, 8, 9. Peptides from some spots, particularly those containing alpha-gliadins, were assigned to multiple proteins that did not have clear matches with Butte 86 sequences. In these cases, the MS/MS data from the spot was further inspected as described [12]. Each peptide identified within a given spot was used to search against the sequences of Butte 86 proteins within the protein group. Peptides that were unique for single Butte 86 proteins are shown in red in Additional files 5, 6, 7, 8, 9. Peptides found in multiple Butte 86 sequences were assigned to Butte 86 proteins for which unique peptides were identified. Butte 86 sequences that accounted for all of the peptides within a given spot were selected as the final identifications and are reported in Additional files 5, 6, 7, 8, 9. In some cases, non-standard chymotryptic or thermolytic cleavages were allowed.

### Cultivar specific sequences

Procedures to develop a set of contigs representing gamma-gliadins and alpha-gliadins for the variety Butte 86 [12,13] were also used to develop contig sets from Butte 86 ESTs that encoded LMW-GS, omega-gliadins, beta-amylases, farinins, purinins and globulins. Sequences of proteins encoded by these unique Butte 86 contigs are included as Additional files 2, 3.

## Abbreviations

2-DE: 2-dimensional gel electrophoresis; ESI: electrospray ionization; EST: expressed sequence tag; HMW: high molecular weight; LMW: low molecular weight; MALDI-TOF: matrix-assisted laser desorption/ionization time of flight mass spectrometry; MS/MS: tandem mass spectrometry; NCBI: National Center for Biotechnology Information; RP-HPLC: reverse phase-high pressure liquid chromatography; SDS-PAGE: SDS polyacrylamide gel electrophoresis; TaGI: Triticum aestivum Gene Index.

## Competing interests

The authors declare that they have no competing interests.

## Authors' contributions

FD was responsible for data analysis and interpretation and drafted the manuscript; WV was responsible for mass spectrometry and database management; CT carried out the quantitative 2-DE gel analysis and spot digestions; WH participated in the 2-DE gel analysis; SA was responsible for DNA sequence analysis and contributed to data analysis and interpretation and manuscript editing. FD and SA contributed equally. All authors read and approved the final manuscript.

## Supplementary Material

Additional file 1 **Supplementary Table 1**. Excel spreadsheet summarizing spot data. Click here for file

Additional file 2 **Word document listing all Butte 86 contig sequences added to the Superwheat database**. Click here for file

Additional file 3 **Word document listing additional Butte 86 contig sequences not in the Superwheat database but used for subsequent manual evaluation**. Click here for file

Additional file 4 **Excel workbook summarizing the Scaffold data for spots 1-100**. Click here for file

Additional file 5 **Excel workbook summarizing the Scaffold data for spots 101 to 200**. Click here for file

Additional file 6 **Excel workbook summarizing the Scaffold data for spots 201 to 300**. Click here for file

Additional file 7 **Excel workbook summarizing the Scaffold data for 301 to 400**. Click here for file

Additional file 8 **Excel workbook summarizing the Scaffold data for spots 401 to 500**. Click here for file

Additional file 9 **Excel workbook summarizing the Scaffold data for spots 501 to 600**. Click here for file
