# Supplementary material for: Deciphering the complexities of the wheat flour proteome using quantitative two-dimensional electrophoresis, three proteases and tandem mass spectrometry
Source: Proteome Sci. 2011 Feb 11;9:10. doi: 10.1186/1477-5956-9-10 (PMC3238214; doi:10.1186/1477-5956-9-10)
Supplement: Additional file 2 — Word document listing all Butte 86 contig sequences added to the Superwheat database. [file 1477-5956-9-10-S2.DOC]

Additional file 2. Protein sequences deduced from Butte 86 contigs that were included in the SuperWheat database. The protein name in black is the designation that appears in Scaffold records, the name in red is the final designation that appears in the text and tables. Gamma-gliadin and alpha-gliadin sequences are described in detail in [12,13].

>BU_Gamma#1(gamma-gliadin Bu-1)

MKTLLILTIIAVALTTTTANIQVDPSGQVQWPQQQQPFPQPQQPQQIFPQPQQTFPHQPQQAFPQPQQTFPHQPQQQFPQPQQPQQPFPQQPQQQFPQPQQPQQPFPQQPQQQFPQPQQPQQPFPQPQQPQLPFPQQPQQPFPQPQQPQQPFPQLQQPQQPLPQPQQPQQPFPQQQQPLIQPYLQQQMNPCKNYLLQQCNPVSLVSSLVSMILPRSDCKVMRQQCCQQLAQIPQQLQCAAIHGVVHSIIMQQEQQQQQQQQQGIQIMRPLFQLVQGQGIIQPQQPAQLEVIRSLVLGTLPTMCNVFVPPECSTTKAPFASIVADIGGQ

>BU_Gamma#2(gamma-gliadin Bu-2)

MKTLLILTILAMAITIGTANIQVDPSGQVQWLQQQLVPQLQQPLSQQPQQTFPQPQQTFPHQPQQQVPQPQQPQQPFLQPQQPFPQQPQQPFPQTQQPQQPFPQQPQQPFPQTQQPQQPFPQQPQQPFPQTQQPQQPFPQLQQPQQPFPQPQQQLPQPQQPQQSFPQQQRSFIQPSLQQQLNPCKNILLQQCKPASLVSSLWSIIWPQSDCQVMRQQCCQQLAQIPQQLQCAAIHSVVHSIIMQQQQQQQQQQGMHIFLPLSQQQQVGQGSLVQGQGIIQPQQPAQLEAIRSLVLQTLPSMCNVYVPPECSIMRAPFASIVAGIGGQ

>BU_Gamma#3(gamma-gliadin Bu-3)

MKTLLILTILAMATTIATANMQVDPSGQVQWPQQQPFPQPQQPFCQQPQRTIPQPHQTFHHQPQQTFPQPQQTYPHQPQQQFPQTQQPQQPFPQPQQTFPQQPQLPFPQQPQQPFPQPQQPQQPFPQSQQPQQPFPQPQQQFPQPQQPQQSFPQQQQPAIQSFLQQQMNPCKNFLLQQCNHVSLVSSLVSIILPRSDCQVMQQQCCQQLAQIPQQLQCAAIHSVAHSIIMQQEQQQGVTILRPLFQLAQGLGIIQPQQPAQLEGIRSLVLKTLPTMCNVYVPPNCSTINVPYANIDAGIGGQ

>BU_Gamma#4(gamma-gliadin Bu-4)

MKTLLIVTILAMATTIATANMQVDPGYQVHWPQQQPFPQPQQPFCQQPQQTIPQPHQTFHHQPQQTFPQPQQTYPHQPQQQFPQTQQPQQPFPQPQQTFPQQPQLPFPQQPQQPFPQPQQPQQQFPQSQQPQQPFPQPQQQFLQPQQPQQSFPQQQQPLIQLSLQQQMNPCKNFLLQQCNPVSLVSSLISMILPRSDCQVMQQQCCQQLAQIPQQLQCAAIHSVVHSIIMQQEQRQGVQIRRPLFQLVQGQGIIQPQQPAQLEVIRSLVLRTLPTMCNVYVSPDCSTINAPFASIVVGIGGQ

>BU_Gamma#5(gamma-gliadin Bu-5)

MKTLLILTILAMAITISTANMQVDPSGQVQWPQQQLVPQPQQPLSQQPQQAFPQPQQTFPHQPQQQVPQPQQPQQPFLQPQQAFPQQPQQPFPQTQQPQQPFPQQPQQPFPQTQQPQQPFPQQPQQPFPQQPQQPFPQTQQPQQPFPQQPQQPFPQTQQPQQPFPQFQQPHQPFHQPQQQFPQPQQPQQSFPQQQRPFIQPSLQQRLNPCKNILLQQCKPASLVSSLWSIIWPQSDCQVMQQQCCQELAQIPQQLQCAAIHSVVHSIIVQQQQQQQQQQQQQQQGMHILLPLSQQQQLGQGTLVQGQGIIQPQQLAQLEAIRSLVLQTLPTMCNVYVPPECSIIRAPFASIVAGIGGQ

>BU_Gamma#6(gamma-gliadin Bu-6)

MKTLFILTILAMATTIATANMQVDPSGQVQWPQQQPFRQPQQPFYQQPQQTFPQPQQAFPHQPQQQFPQPQQPQQQFPQPQQPQQPFPQPQQAQLPFPQQPQQPFPQPQQPQQPFPQSQQPQQPFPQPQQPQQSFPQQQQPLIQPYLQQQMNPCKNYLLQQCNPVSLVSSLVSMILPRSDCQVMQQQCCQQLAQIPRQLQCAAIHSVVHSIVMQQEQQQGIQILRPLFQLVQGQGIIQPQQPAQYEVIRSLVLRTLPNMCNVYVRPDCSTINAPFASIVAGISGQ

>BU_Gamma#7(gamma-gliadin Bu-7)

MKTLLILTILAMAITIATANMQVDPSGQVQWPQQQPFLQPHQPFSQQPQQIFPQPQQTFPHQPQQQFPQPQQPQQQFLQPRQPFPQQPQQPYPQQPQQPFPQTQQPQQPFPQSKQPQQPFPQPQQPQQSFPQQQPSLIQQSLQQQLNPCKNFLLQQCKPVSLVSSLWSIILPPSDCQVMRQQCCQQLAQIPQQLQCAAIHSVVHSIIMQQEQQEQLQGVQILVPLSQQQQVGQGILVQGQGIIQPQQPAQLEVIRSLVLQTLPTMCNVYVPPYCSTIRAPFASIVASIGGQ

>BU_Gamma#8 (gamma-gliadin Bu-8)

MKTLLILTILAMATTIATANMQVDPSGQVQWPQQQPFPQPQQPFCQQPQRTIPQPHQTFHHQPQQTFPQPQQTFPHQPQQQFPQPQQPQQPFPQQPQQQFPQPQQPQQPFPQQPQQQFPQPQQPQQPFPQPQQPQLPFPQQPQQPFPQPQQPQQPFPQLQQPQQPLPQPQQPQQPFPQQQQPLIQPYLQQQMNPCKNYLLQQCNPVSLVSSLVSMILPRSDCKVMRQQCCQQLAQIPQQLQCAAIHGVVHSIIMQQEQQQQQQQQQGIQIMRPLFQLVQGQGIIQPQQPAQLEVIRSLVLGTLPTMCNVFVPPECSTTKAPFASIVADIGGQ

>BU_Gamma#9 (gamma-gliadin Bu-9)

MKTLLILTILAMATTIATANMQVDPSSRVQWPQEQPPPQSQQPFSQQPQQIFPQPQQTFPHQPQQAFLQPQQTFPRRPQQQFPQPQQPQQPFPQPQQPQLPFPQQPQQPFPQPQQPQQPFPQSQQPQQPFPQPQQQFPQPQQPQQSFPQQQQWMIQSFLQQQMNPCKNFLLQQCNPVSLVSSLVSIILPRSDCQLMQQQCCQQLAQIPQQLQCAAIHNVAHSIIMQQEQQRGVQILRPLFQLAQGLGIIQPQQPAQLEGIRPLVLKTLPTMCNVYVPPDCSTINVPYAS

>BU_Gamma#10 (gamma-gliadin Bu-10)

MKTLLILTILAMATTIATANMQVDPSGQVQWPQQQPFPQPQQPFCQQPQRTIPQPHQTFHHQPQQFPQTQQPQQPFPQPQQTFPQQPQLPFPQQPQQPFPQPQQPQQPFPQSQQPQQPFPQPQQQFPQPQQPQQSFPQQQQPAIQSFLQQQMNPCKNFLLQQCNHVSLVSSLVSIILPRSDCQVMQQQCCQQLAQIPQQLQCAAIHSVAHSIIMQQEQQQGVTILRPLFQLAQGLGIIQPQQPAQLEGIRSLVLKTLPTMCNVYVPPNCSTINVPYANIDAGIGGQ

>BU_Gamma#11 (gamma-gliadin Bu-11)

PQQSFPQQQPPFIQPSLQQQVNPCKNFLLQQCKPVSLVSSLWSMIWPQSDCQVMRQQCCQQLAQIPQQLQCAAIHTVIHSIIMQQEQQQGMHILLPLYQQQQVGQGTLVQGQGIIQPQQPAQLEAIRSLVLQTLPTMCNVYVPPECSIIKAPFSSVVAGIGGQ

>BU_Alpha#1 (alpha-gliadin Bu-1)

MKTFLILALLAIVATTATIAVRVPVPQLQPQNPSQQQPQEQVPLVQQQQFPGQQQPFPPQQPYPQPQPFPSQQPYLQLQPFPQPQLPYPQPQLPYPQPQLPYPQPQPFRPQQPYPQSQPQYSQPQQPISQQQQQQQQQQQKQQQQQQQQQILQQILQQQLIPCRDVVLQQHSIAYGSSQVLQQSTYQLVQQLCCQQLWQIPEQSRCQAIHNVVHAIILHQQQQQQQQQQQKQPLSQVSFQQPQQQYPSGQGSFQPSQQNPQAQGSVQPQQLPQFEEIRNLALETLPAMCNVYIPPYCTIAPVGIFGTN

>BU_Alpha#2(alpha-gliadin Bu-2)

MKTFLILALLAIVATTATIAVRVPVPQLQPQNPSQQQPQKQVPLVQQQQFPGQQQPFPPQQPYPQLQPFPSQQPYMQLQPFPQPQLPYPQPQLPYPQPQPFRPQQSYPQPQPQYSQPQQPISQQQQQQQQQQQILQQILQQQLIPCRDVVLQQHSIAHGSSQVLQQSTYQLVQQLCCQQLWQIPEQSRCQAIHNVVHAIILHQQQQQQQQQPLSQVCFQQSQQQYPSGQGSFQPSQQNPQAQGSVQPQQLPQFEEIRNLALETLPAMCNVYIPPYCTIAPVGIFGTN

>BU_Alpha#3(alpha-gliadin Bu-3)

MKTFLILALLAIVATTATIAVRVPVPQLQPQNPSQQQPQEQVPLVQQQQFPGQQQPFPPQQPYPQPQPFPSQQPYLQLQPFPQPQLPYPQPQLPYPQPQLPYPQPQPFRPQQPYPQSQPQYSQPQQPISQQQQQQQQQQQQKQQQQQQQQILQQILQQQLIPCRDVVLQQHSIAYGSSQVLQQSTYQLVQQLCCQQLWQIPEQSRCQAIHNVVHAIILHQQQQQQQQQQQPLSQVSFQQPQQQYPSGQGSFQPSQQNPQAQGSVQPQQLPQFEEIRNLALETLPAMCNVYIPPYCTIAPVGIFGTN

>BU_Alpha#4(alpha-gliadin Bu-4)

MKTFLILALLAIVATTATIAVRVPVPQLQPQNPSQQQPQEQVPLVQQQQFPGQQQPFPPQQPYPQPQPFPSQQPYLQLQPFPQPQLPYPQPHLPYPQPQPFRPQQPYPQPQPQYSQPQQPISQQQQQQQQQQQQQILQQILQQQLIPCRDVVLQQHSIAHGSSQVLQQSTYQLVQQLCCQQLWQIPEQSRCQAIHNVVHAIILHQQQQQQQQQQQQPLSQVSFQQPQQQYPSGQGSFQPSQQNPQAQGSVQPQQLPQFEEIRNLALETLPAMCNVYIPPYCTIAPVGFFGTN

>BU_Alpha#5(alpha-gliadin Bu-5)

MKTFLILALLAIVATTATTAVRVPVPQLQPQNPSQQQSQEQVPLVQQQQFLGQQQPFPPQQPYPQPQPFPSQQPYLQLQPFPQPQLPYSQPQPFRPQQPYPQPQPQYSQPQHPISQQQQQQQQQQQQEQQILQQILQQQLIPCMDVVLQQHNIAHGRSQVLQQSTYQLLQELCCQHLWQIPEQSQCQAIHNVVHAIILRQQQKQQQQPSSQVSFQQPQQQYPLGQGSFRPSQQNPQAQGSVQPQQLPQFEEIRNLALQTLPAMCNVYIPPYCTIAPFGIFGTN

>BU_Alpha#6(alpha-gliadin Bu-6)

MKTFLIISLLAIVATTATTAVRVPVPQLQPQNPSLQQPQEQVPLVQQQQFPGQQQTFPPQQPYPQPQPFPAQQPYPQPQLFPQPQPFPPQLPYPQPQPFPPQQPYPQPQTQHLQPQQPISQQQAQQQQQQQQQQQILQQILQQQQLIPCRDVIVLQQHNIAHESSQVLQQSSYQVLQQLCCQQLRQIPEQSRCQAINNVVHAIILHQQQQQQGQHQQSSQVSYQQPQQQYPSGQGSFQPSQQNPQAQGFVQPQHL

>BU_Alpha#8(alpha-gliadin Bu-8)

MKTFLILALLAIVATTATTAVRVPVPQLQPQNPSQQQPQEQVPLVQQQQFPGQQQQFPPQQPYPQPQPFPSQQPYLQLQPFPQPQPFPPQLPYPQPQSFPPQQPYPQQQPQYLQPQQPISQQQAQQQQQQQQQQQQQQQILQQILQQQLIPCRDVVLQQHNIAHASSQVLQQSTYQLLQQLCCQQLLQIPEQSRCQAIHNVAHAIIMHQQQQQQQEQQQQLQQQQQQQLHQQRQQPSSQVSFQQPQQQYPSSQVSFQPSQLNPQAQGSVQPQQLPQFAEIRNLALQMLPAMCNVYIPPHCSTTIAPFGIFGTN

>BU_Alpha#9(alpha-gliadin Bu-9)

QPYPQPQLFPQPQPFPPQLPYPKPQPFPPQQPYPQPQTQHLQPQQPISQQQAQQQQQQQQQILQQILQQILQQQQLIPCRDVIVLQLHNIAHESSQVLQQSSYQVLQQLCCQQLWQIPELSRCQAIHNVVHAIILHQQQQQQEQEQHQPSSQVSYQQPQQQYPSGQGSFQPSQQNPQAQGFVQPQRLPQLKEIRNLALQTLPAMCNVYIPPYCSTTIAPFGIFGTN

>BU_Alpha#10(alpha-gliadin Bu-10)

MKTFLILALLAIVATTATTAVRVPVPQLQLQNPSQQQPQEQVPLVQEQQFPGQQQPFPPQQPYPQPQPFPSQQPYLQLQPFPQPQLPYPQPQPFRPQQPYPQPQPQYSQPQQPISQQQQQQQQQQQQQQQILQQILQQQLIPCRDVVLQQHNIAHGSSQVLQESTYQLVQQLCCQQLWQIPEQSRCQAIHNVVHAIILHQQHHHHHQQQQQQQQQPLSQVSFQQPQQQYPSGQGFFQPSQQNPQAQGSFQPQQLPQFEAIRNLALQTLPAMCNVYIPPYCTIAPFGIFGTN

>BU_Alpha#11(alpha-gliadin Bu-11)

MKTFLIISLLAIVATTATTAVRVPVPQLQPQNPSLQQPQEQVPLVQQQQFPGQQQTFPPQQPYPQPQPFPGQQPYPQPQLFPQPQPFPPQLPYPQPQPFPPQQPYPQPQTHHLQPQQPISQQQVQQQQQQQQQQQQQQQQQQQQQQLLLQQQILQQILQQQQLIPCRDVIVLQQHNIAHESSQVLQQSSYQVLQQLCCQQLRLIPEQSRCQAIHNVVHAIILHQQQQQQQQQQQQQQGQHQPSSQVSYQQPQQQYPSGQGSFQPSQQNPQAQGFVQPQHLPQLEEISNLALQTLPAMCNVYIPPYCSTTIAPFGIFGTN

>BU_Alpha#12(alpha-gliadin Bu-12)

ALLAIVATTTTTAVRVPVPQLQPQNPSQQQPQEQVPLVQQQQFLGQQQQQFPGQQQPFPPQQPYPQPQPFLPQLPYPQPQPFPPQQSYPQPQPQYPQPQQPISQQQAQLQQQQQQQQQQQQILQQILQQQLIPCRDVVLQQPNIAHASSKVSQQSYQLLQQLCCQQLWQTPEQSRCQAIHNVIHAIILHQQQQQQQQQQQQQQQQQQPSSQVSYQQPQQQYPSGQGFFQPSQQNPQAQGFVQPQQLPQFEEIRNLALQTLPAMCNVYIPPYCSTTIAPFGIMSTN

>BU_Alpha#13(alpha-gliadin Bu-13)

MKTFLILALLAIVATTATTAVRVPVPQLQLQNPSQQQPQEQVPLVQEQQFPGQQQPFPPQQPYPQPQPFPSQQPYLQLQPFPQPQQPYPQQQPQYLQPQQPISQQQAQQQQQQQQQQQQQQQILQQILQQQLIPCRDVVLQQHNIAHASSQVLQQSTYQLLQQLCCQQLLQIPEQSRCQAIHNVAHAIIMHQQQQQQQEQQQQLQQQQQQQLHQQRQQPSSQVSFQQPQQQYPSSQVSFQPSQLNPQAQGSVQPQQLPQFAEIRNLALQMLPAMCNVYIPPHCSTTIAPFGIFGTN

>BU_Alpha#14(alpha-gliadin Bu-14)

MKTFLILALLAIVATTATTAVRVPVPQLQPQNPSQQQPQEQVPLVQQQQFLGQQQPFPPQQPYPQPQPFPSQQPYLQLQPFPQPQLPYSQPQPFRPQQPYPQPQPQYSQPQEPISQQQQQQQQQQQILQQILQQQLIPCMDVVLQQHNIAHGRSQVLQQSTYQLLQELCCQHLWQIPEQSQCQAIQNVVHAIILHQQQKQQQQPSSQVSFQQPLQQYPLGQGSFRPSQQNPQDQGSVQPQQLPQFEEIRNLALQTLPAMCNVYIPPYCTIAPFGIFGTN

>BU_Alpha#15(alpha-gliadin Bu-15)

QPQQPISQQQAQQQQQQQQILQQILQQQLIPCRDVVLQQHNIAHASSQVLQQSSYQQLQQLCCQQLFQIPEQSRCQAIHNVVHAIILHHHQQQQQQPSSQVSYQQPQEQYPSGQGSFQSSQQNPQAQGSVQPQQLPQFQEIRNLALQTLPAMCNVYIPPYCSTTIAPFGIFGTN

>BU_Alpha#16(alpha-gliadin Bu-16)

MKTFLILALLAIVATTTTTAVRVPVPQLQPQNPSQQQPQEQVPLVQQQQFLGQQQQHFPGQQQPFPPQQPYPQPQPFLPQLPYPQPQPFPPQQSYPQPQPQYPQPQQPISQQQAQLQQQQQQQQQQILQQILQQQLIPCRDVVLQQPNIAHASSQVSQQSYQLLQQLCCQQLWQTPEQSRCQAIHNVIHAIILHHQQQQQQQQQQQQQQQQQQQQQQQQ

>BU_Alpha#17(alpha-gliadin Bu-17)

IRHEAIIPATRTNNFPATPQPQYPQPQQPISQQQAQLQQQQQQQQQQQILQQILQQQLIPCRDVVLQQPNIAHASSQVSQQSYQLLQQLCCQQLWQTPEQSRCQAIHNVIHAIILHQQQQQQQQQQQQQQQQQQQQQQQQQQPSSQVSYQQPQQQYPSGQGFFQPSQQNPQAQGFVQPQQLPQFEEIRNLALQTLPAMCNVYIPPYCSTTIAPFGIMSTN

>BU_Alpha#18(alpha-gliadin Bu-18)

LHQQQQRQQQQQQQQQQQQQQQQQQQQQQQQQQQQQQQQQQPSSQVSFQQPQQQYPSGQGSFQPSQQNPQAQSSVQHQQLPQFEEIRNLALQTLPAVCNVYIPPYCSTTIAPFGIFGTN

>BU_Alpha#23(alpha-gliadin Bu-23)

MKTFLILALLAIVATTATTAVRVPVPQLQPQNPSQQQPQEQVPLVQQQQFPGQQQQFPPQQPYPQPQPFPSQQPYLQLQPFPQPQPFPPQLPYPQPQSFPPQQPYQQQQPQYLQPQQPISQQQAQQQQQQQQQQQQQQILQQILQQQLIPCRDVVLQQHNIAHASSQVLQQSTYQLLQQLCCQQLLQIPEQSRCQAIHNVAHAIIMHQQQQQQQEQQQQLQQQQQQQLQQQRQQPSSQVSFQQPQQQYPSSQVSFQPSQLNPQAQGSVQPQQLPQFAEIRNLALQTLPAMCNVYIPPHCSTTIAPFGIFGTN

>BU_Alpha#26(alpha-gliadin Bu-26)

MKTFLILALLAIVATTATIAVRVPVPQLQPQNPSQQQPQEQVPLVQQQQFPGQQQPFPPQQPYPQLQPFPSQQPYMQLQPFPQPQLPYPQPQLPYPQPQPFRPQQSYPQPQPQYSQPQQPISQQQQQQQQQQQQQILQQILQQQLIPCRDVVLQQHSIAHGSSQVLQQSTYQLVQQLCCQQLWQIPEQSRCQAIHNVVHAIILHQQQQQQQQQQQQQQQQQ

>BU_Omega#D1(omega-gliadin Bu-D1)

FPQQPQQPFPQPQQPIPVQPQQSFPQQSQQSQQPFAQPQQLFPELQQPIPQQPQQPFPLQPQQPFPQQPQQPFPQQPQQSFPQQPQQPFPQQPQQPFPQQPQQPFPQQPQQPFPLRPQQPFPQQPQQSQQSFPQPQPQQPQQPSILQPQQPLPQQPQQPFQQPQQQLSQQPEQTISQQPQQPFPQQPHQPQQPYPQQQPYGSSLTSIGGQ

>BU_Omega#D2(BQ804665)(omega-gliadin Bu-D2)

PQQPYPQQPYPSQQPYPSQQPFPTPQPQFPQQSQQPFTQPQQPTPLQPQQPFPQQPQQPQQPFPQPQQPFPWQPQQPFPQTQQSFPLQPQQPFPQQPQQPFPQPQLQFPQQPEQIIPQQPQQPFLLESQQPFPQQPQQPFPQPQQLIPMQPQQPFPQQSQQSQQPFPGPQQLFPELQQPIPQQPQQPFPL

>BU_Omega#D3(BQ838934)(omega-gliadin Bu-D3)

PFPQQPQQPFPQPQLPFPQQSEQIIPQQPQQPFPLQPQQPFPQQPQQPFPQPQQPIPVQPQQSFPQQSQQSQQPFAQPQQLFPELQQPIPQQPQQPFPLQPQQPFPQQPQQPFPQQPQQSFPQQPQQPFPQQPQQPYPQQQPYGTSLTSIGGQ

>BU_Omega#D4(BQ804424)(omega-gliadin Bu-D4)

PQQPQQPFPQTQQPQQPFPQLQQPQQPIPQQPQQPFPLQPQQPFPQQSQQPFPQQPQQPCPLQPQQPFPQQPQQPFPQQPQQPFPLQPQQPFPLRPQQPFSQQPQQSQQSFPQPQPQQPQQPSILQPQQPFLQPQQQLSQQLEQTISQQPQQPFPQQPHQPQQPYPQQQPY

>BU_Omega#D5(BQ805896)(omega-gliadin Bu-D5)

QPQQPQQPSILQPEQPLPQRPQQPFLLPQQQLSQQPEQTISQQPQQPHQPQQPYPQQQPYGTSLTSIGGQ

>BU_Omega#B1)(omega-gliadin Bu-B1)

FPQQEFPQQQQFPQQQIAQQPQQLPQQQQIPQQPQQFPQQQQFPQQQSPQQQQFPQQQFPQQQQLPQQQFPQPQQIPQQQQIPQQPQQFPQQQFPQQQQFPQQQEFPQQQFPQQQFHQQQLPQQQFPQQQFPQQQFPQQQQFPQQQQLTQQQFPRPQQSPEQQQFPQQQFPQQPPQQFPQQQFPIPYPPQQSQEPSPYQQYPQQQPSGSDVISISGL

>BU_Omega#B2(omega-gliadin Bu-B2)

PQQQIPQQPQQFPQQQFPQQQQFPQQQEFPQQQFPQQQFHQQQFPQQQFPQQQFPQQQQFPQQQQLTQQQFPRPQQSPEQQQFPQQPPQQFPQQQFPIPYPPQQSQEPSPYQQYPQQQPSGSDVISISGL

>BU_Omega#B3(BQ806240)(omega-gliadin Bu-B3)

IPQQPQQFLQQQQFPQQQPPQQHQFPQQQLPQQQQIPQQPQQIPQQQQIPQQPQQFPQQQFPQQQFPQQQFPQQEFPQQQQFPQQQIAQQPQQLPQHQQIPQQPQQFPQQQQFPQQQSPQQQQFPQQQFPQQQQLPQKQFPQPQQIPQQQQIPQQPQQFPQQQFPQQQQFPQQQEFPQQQFPQQQFHQQQFPQQQFPQQ

>BU_LMW-GS#1 (LMW-GS Bu-1)

MKTFLVFALLAVAATSAIAQMETRCIPGLERPWQQQPLPPQQTFPQQPLFSQQQQQQLFPQQPSFSQQQPPFWQQQPPFSQQQPILPQQPPFSQQQQLVLPQQPPFSQQQQPVLPPQQSPFPQQQQQHQQLVQQQIPVVQPSILQQLNPCKVFLQQQCSPVAMPQRLARSQMLQQSSCHVMQQQCCQQLPQIPQQSRYEAIRAIIYSIILQEQQQVQGSIQSQQQQPQQLGQCVSQPQQQSQQQLGQQPQQQQLAQGTFLQPHQIAQLEVMTSIALRILPTMCSVNVPLYRTTTSVPFGVGTGVGAY

>BU_LMW-GS#2(LMW-GS Bu-2)

QQQQPVLPQQPSFSQQQLPPFSQQQPPFSQQQQPVLPQQPPFSQQQQPILPQQPPFSQQQQQPVLPQQQILFVHPSILQQLNPCKVFLQQQCSPVAMPQSLARSQMLQQSSCHVMQQQCCQQLLQIPQQSRYEAIRAIIYSIILQEQQQVQGSIQTQQQQPQELGQCVSQPQQQSQQQLGQQPQQQQLAQGTFLQPHQIAQLEVMTSIALRTLPTMCRVNVPLYRTTTSVPFGVGAGVGAY

>BU_LMW-GS#3(LMW-GS Bu-3)

LPPFSQQQQPVLPQQPPFSQQQQQPILPQQPPFSQQQQPVLLQQQIPFVHPSILQQLNPCKVFLQQQCSPVAMPQSLARSQMLQQSSCHVMQQQCCQQLPQIPQQSRYEAIRAIVYSIILQEQQQVQGSIQTQQQQPQQLGQCVSQPQQQSQQQLGQQPQQQQLAQGTFLQPHQIAQLEVMTSIALRTLPTMCNVNVSLYRTTTRVPFGVGTGVGGY

>BU_LMW-GS#4(LMW-GS Bu-4)

QQQQPPFSQQQQPQFSQQQQPPYSQQQQPPYSQQQQPPFSQQQQPPFSQQQQQPPFTQQQQQQQQQQPFTQQQQPPFSQQPPISQQQQPPFLQQQRPPFSRQQQIPVIHPSVLQQLNPCKVFLQQQCIPVAMQRCLARSQMLQQSICHVMQQQCCQQLRQIPEQSRHESIRAIIYSIILQQQQQQQQQQQQQQGQSIIQYQQQQPQQLGQCVSQPLQQLQQQLGQQPQQQQLAHQIAQLEVMTSIALRTLPTMCNVNVPLYETTTSVPLGVGIGVGVY

>BU_LMW-GS#5(LMW-GS Bu-5)

MKTFLIFALLAIAATSAIAQMETSRVPGLEKPWQQQPLPPQQQPPCSQQQQPFPQQQQPIIILQQSPFSQQQQPVLPQQPPFSQQQQPPFSQQQQPSSQQPPFPQQHQQFPQQQIPVVQPSVLQQLNPCKVFLQQQCSHVAMSQRLARSQMWQQSSCHVMQQQCCQQLPQIPEQSRSEAIRAIVYSIILQEQQQGFVQPQQQQPQQSGQGVSQHQQQSQQQQQLGQCSFQQPQQLQQLGQQPQQQQIPQGIFLQPHQISQLEVMTSIALRTLPTMCGVNVPLYSSTTIMPFSIGTGVGGY

>BU_LMW-GS#6(LMW-GS Bu-6)

MKTFLVFALLAVVATSAIAQMETSCIPGLERPWQQQPLQQKETFPQQPPSSQQQQPFPQQPPFLQQQPSFSQQPLFSQKQQPVLPQQPAFSQQQQTVLPQQPAFSQQQHQQLLQQQIPIVHPSILQQLNPCKVFLQQQCSPVAMPQHLARSQMWQQSSCNVMQQQCCQQLPRIPEQSRYEAIRAIIFSIILQEQQQGFVQPQQQQPQQSVQGVYQPQQQSQQQLGQCSFQQPQQQLGQQPQQQQVQKGTFLQPHQIARLEVMTSIALRTLPTMCSVNVPLYSSITSAPLGVGSRVGAY

>BU_LMW-GS#7(LMW-GS Bu-7)

MKTFLIFALLAIAATSAIAQMETSRVPGLEKPWQQQPLPPQQQPPCSQQQQPFPQQQQPIIILQQSPFSQQQQPVLPQQQPVIILQQPPFSQQQQPVLPQQPPFSQQQQQQQQQQPPFSQQQQPVLPQQPPFSQQQQPPFSQQQQPSSQQPPFPQQHQQFPQQQIPVVQPSVLQQLNPCKVFLQQQCSHVAMSQRLARSQMWQQSSCHVMQQQCCQQLPQIPEQSRSEAIRAIVYSIILQEQQQGFVQPQQQQPQQSGQGVSQHQQQSQQQQQLGQCSFQQPQQLQQLGQQPQQQQIPQGIFLQPHQISQLEVMTSIALRTLPTMCGVNVPLYSSTTIMPFSIGTGVGGY

>BU_LMW-GS#8(LMW-GS Bu-8)

MKTFLVFALIAVVATSAIAQMETSCISGLERPWQQQPLPPQQSFSQQPPFSQQQQQPLPQQPSFSQQQPPFSQQQPILSQQPPFSQQQQPVLPQQSPFSQQQQLVLPPQQQQQQLVQQQIPIVQPSVLQQLNPCKVFLQQQCSPVAMPQRLARSQMWQQSSCHVMQQQCCQQLQQIPEQSRYEAIRAIIYSIILQEQQQGFVQPQQQQPQQSGQGVSQSQQQSQQQLGQCSFQQPQQQLGQQPQQQQQQVLQGTFLQ

>BU_LMW-GS#10(BQ804218)(LMW-GS Bu-10)

MKTFLIFALLAIAATSAIAQMETSRVPGLEKPWQQQPLPPQQQPPCSQQQQPFPQQQQPIIILQQSPFSQQQQPVLPQQQPVIILQQPPFSQQQQPVLPQQPPFSQQQQPPFSQQQQPSSQQPPFPQQHQQFPQQQIPVVQPSVLQQLNPCKVFLQQQCSHVAMSQRLARSQMWQQSSCHVMQQQCCQQLPQIPEQSRSEAIRAIVYSIILQEQQQGFVQPQQQQPQQSGQGVSQHQQQSQQQQ

>BU_LMW-GS#11(LMW-GS Bu-11)

ARGQQPPFSQQRPPFSQQQQQPVLPQQPPFSQQQQQQPILPQQPPFSQHQQPVLPQQQIPYVQPSILQQLNPCKVFLQQQCSPVAMPQSLARSQMLWQSSCHVMQQQCCQQLPRIPEQSRYDAIRAIIYSIVLQEQQHGQGFNQPQQQQPQQSVQGVSQPQQQQKQLGQCSFQRPQQQQLGQWPQQQQVPQGTLLQPHQIAQLELMTSIALRTLPMMCSVNVPVYGTTTS

>BU_LMW-GS#12(BQ804627)(LMW-GS Bu-12)

LPQQQPVIILQQPPFSQQQQPVLPQQPPFSQQQPPFSQQQQPVLPQQPPFSQQQQPILPQQPPFSQQQQQPVLPQQQILFVHPSILQQLNPCKVFLQQQCSPVAMPQSLARSQMLQQSSCHVMQQQCCQQLLQIPQQSRYEAIRAIIYSIILQEQQQVQGSIQTQQQQPQELGQCVSQPQQQSQQQLGQQPQQQQLAQGTFLQPHQIAQLEVMTSIALRTLPTMCRVNVPLYRTTTSVPFGVGAGVGAY

>BU_LMW-GS#13(LMW-GS Bu-13)

MENSHIPGLERPSQQQQPVLPQQPPFSQQQQPILPQQPPFSQQQQQPVLPQQQILFVHPSILQQLNPCKVFLQQQCSPVAMPQSLARSQMLQQSSCHVMQQQCCQQLLQIPQQSRYEAIRAIIYSIILQEQQQVQGSIQTQQQQPQELGQCVSQPQQQSQQQLGQQPQQQQLAQGTFLQPHQIAQLEVMTSIALRTLPTMCRVNVPLYRTTTSVPFGVGAGVGAY

>BU_LMW-GS#14(LMW-GS Bu-14)

MKTFLIFALLAIAATSAIAQMETSRVPGLEKPWQQQPLPPQQQPPCSQQQQPFPQQQQQQQQQQPPFSQQQQPVLPQQPPFSQQQQPPFSQQQQPSSQQPPFPQQHQQFPQQQIPVVQPSVLQQLNPCKVFLQQQCSHVAMSQRLARSQMWQQSSCHVMQQQCCQQLPQIPEQSRSEAIRAIVYSIILQEQQQGFVQPQQQQPQQSGQGVSQHQQQSQQQQQLGQCSFQQPQQLQQLGQQPQQQQIPQGIFLQPHQISQLEVMTSIALRTLPTMCGVNVPLYSSTTIMPFSIGTGVGGY

>BU_LMW-GS#17(LMW-GS Bu-17)

MKTFLIFALLAIAATSAIAQMETSRVPGLEKPWQQQPLPPQQQPPCSQQQQPFPQQQQPIIILQQSPFSQQQQPVLPQQQPVIILQQPPFSQQQQPVLPQQPPFSQQQQQPILPQQPPFSQQQQPVLLQQQIPFVHPSILQQLNPCKVFLQQQCSPVAMPQSLARSQMLQQSSCHVMQQQCCQQLPQIPQQSRYEAIRAIVYSIILQEQQQVQGSIQTQQQQPQQLGQCVSQPQQQSQQQLGQQPQQQQGQGSSQPQQQTQLDQGWIAVIGTWVIQTIPAMCDVHVPPYCYTTISPSIDVTTGMGGY

>BU_LMW-GS#18(BQ806669)(LMW-GS Bu-18)

QQPVLPQQQIPSVQPSILQQLNPCKVFLQQQCSPVAMPQSLARSQMLWQSSCHVMQQQCCRQLPQIPEQSRYDAIRAIIYSIVLQEQQHGQGLNQPQQQQPQQSVQGVSQPQQQQKQLGQCSFQQPQQQQLGQWPQQQ.VPQGTLLQPHQIAQLEVMTSIALRTLPTMCSVNVPVYGTTTIVPFGVGTRVGAY

>BU_LMW-GS#20(LMW-GS Bu-20)

QQQIPFVHPSILQQLNPCKVFLQQQCSPVAMPQSLARSQMLQQSSCHVMQQQCCQQLPQIPQQSRYEAIRAIVYSIILQEQQQVQGSIQTQQQQPQQLGQCVSQPQQQSQQQLGQQPQQQQLAQGTFLQPHQIAQLELMTSIALRTLPTMCNVNVPLYRTTTRVPFGVGTGVGGY

>BU_LMW-GS#21(LMW-GS Bu-21)

IILQEQQQVQGSIQTQQQQPQQLGQCVSQPQQQSQQQLGQQPQQQQLAQGTFLQPHQIAQLEVMTSIALRTLPTMCNVNVSLYRTTTRVPFGVGTGVGGY

>BU_LMW-GS#22(BQ805913) (LMW-GS Bu-22)

LPPFSQQQQPVLPQQPPFLQQQLPPFSQQLPPFSQQQQPVLPQQPPFSQQQQQPILPQQPPFSQQQPILPQQPPFSQQQQLVLPQQPPFSQQQQPVLPPQQSPFPQQQQQHQQLVQQQIPVVQPSILQQLNPCKVFLQQQCSPVAMPQRLARSQMLQQSSCHVMQQQCCQQLPQIPQQSRYEAIRAIIYSIILQEQQQVQGSIQSQQQ

>BU_Beta-amylase#1(beta-amylase Bu-1)

MEASAQQGNYVQVYVMLPLDIVSVNNRFEKGDELRGQLKRLVEAGVDGVMVDVWWGLVEGKGPRVYDWSAYKQLFELVHEAGLKLQAIMSFHQCGGNVGDVVNIPIPQWVRNVGVSDPDIFYTDQHGTRNIEYLTLGVDDQPLFHGRSAVQMYADYMASFRDNMKEFLDAGLIVDIEVGLGPAGELRYPSYPQSHGWSFPGIGEFICYDKYLQADFKAAAAMVGHPEWEFPRDAGTYNDTPQRTRFFVDNGTYLTEQGRFFLAWYSNNLIKHGDKILDEANKVFLGHTVQLAIKISGIHWWYKVPSHAAEVTAGYYNLHDRDGYRPIARMLKRHHASLNFTCAEMRDSEQSSQAMSAPEELVQQVLSAGWREGLNMACENALPRYDPTAYNTILRNARPHGINKSGPPEHKLFGFTYLRLSNQLVEGQNYVNFKTFVDRMHANLPHDPCVDPVAPLQRSGPELTIEMILQAAQPKLEPFPFEEHTDLPVQGLGGIGGGEVEDPTGGMGGEVQQDPTGGMGGEVQQDPTGGMGGEVEDP

>BU_Beta-amylase#2(beta-amylase Bu-2)

MEASVQQGNYVQVYVMLPLDAVSVNNRFEKGDELREQLKRLVEAGVDGVMVDVWWGLVEAKGPRAYDWSAYKQLFQLVHEAGLKLKAIMSFHQCGGNVGDVVNIPIPQWVRNVGASDPDIFYTDQHGTRNIEYLTLGVDDQPLFHGRSAVQMYTDYMASFRDNMKEFLDAGVIVDIEVGLGPAGELRYPSYPQSHGWSFPGIGEFICYDKYLQADFKAAAAMVGHPEWEFPRDAGQYNDTPQRTRFFVDNGTYLTEQGRFFLAWYSNNLIKHGDKILDEANKVFLGHRVQLAIKISGIHWWYKVPSHAAEITAGYYNLHDRDGYRPIARMLKRHRASLNFTCAEMRDSEQSSQAMSAPEELVQQVLSAGWREGLNMACENALPRYDPTAYNTILRNARPHGINKSGPPEHKLFGFTYLRLSNQLVEGQNYVNFKTFVDRMHANLPHDPCVDPVAPLQRSGPELTIEMILQAAQPKLGSIPL

>BU_Beta-amylase#3 (beta-amylase Bu-3)

MEASVQQGNYVQVYVMLPLDAVSVNNRFEKGDELRPQLKRLVDAGVDGVMVDVWWGLVEAKGPRVYDWSAYKQLFKLVHEAGLKLQAIMSLHQCGGNVGDVVNIPIPQWVRDVGASDPDIFYTDQHGTRNIEYLTLGVDDQPLFHGRSAVQMYADYMASFRDNMKEFLDAGVIVDIEVGLGPAGELRYPSYPQSHGWSFPGIGEFICYDKYLQADFKAAAAMVGHPEWEFPHDSGTYNDTPERTRFFVDNGTYLTEQGRFFLAWYSNNLIKHGDKILDEANKVFLGHRVQLAIKISGIHWWYKAPSHAAELTAGYYNLHDRDGYRPIARMLKRHHASLNFTCAEMRDSEQSSQARSAPEELVQQVLSAGWREGLNVACENALPRYDPTAYNTILRNARPHGINKSGPPEHKLFGFTYLRLSNQLVEGQNYVNFKTFVDRMHANLPHDPCVDPVAPLQRSGPELTIEMILQAAQPKLEPFP

>BU_ LTP #1 (BQ806332) (LTP Bu-1)

MARAQVMLMAVALVLMLAAVPRAAVAIDCGHVDSLVRPCLSYVQGGPSPSGQCCGGVQSLHNQAQSKSDRQAACNCLKGIARGIHNLNEDNARSLAPKCGVNLPYHISLDIDCNSV

>BU_LTP#2(BQ838835) (LTP Bu-2)

MARAQVMLMAVALVLMLAAVPRAAVAIDCGHVDSLVRPCLSYVQGGPGPSGQCCDGVKNLHNQARSQSDRQSACNCLKGIARGIHNLNEDNARSIPPKCGVNLPYTISLNIDCSRV

>BU_ LTP#3(BQ806793) (LTP Bu-3)

MARLNSKAVAAAVVLAAVVLMMAGREASAALSCGQVDSKLAPCVAYVTGRASSISKECCSGVQGLNGLARSSPDRKIACRCLKSLATSIKSINMGKVSGVPGKCGVSVPFPISMSTNCNNVN

>BU_LTP #4(BQ838992) (LTP Bu-4)

MARLNSKAVVAAVVLAAVVLMMAGREASAALSCGQVDSKLAPCVAYVTGRASSISKECCSGVQGLNGMARSSSDRKIACRCLKSLATSIKSINMGKVSGVPGKCGVSVPFPISMSTNCDTVN

>BU_LTP #5(BQ804735) (LTP Bu-5)

MARLNSKAVVSAVVLAAVVLMMAGREATALSCGQVDSKLAPCVSYVTGKAPSISKECCSGVQGLNGLARSSPDRKIACRCLKSLATSIKSINMDKVSGVPGKCGVSVPFPISMSTNCNNVN

>BU_LTP #6(BQ804678) (LTP Bu-6)

MAAPRGAAVMLAVVLAAMVVAPPATVHAISCSTVYSTLMPCLQYVQQGGSPARGCCTGIQNLLAEANNSPDRRTICGCLKNVANGASGGPYITRAAALPSKCNVALPYKISPSVDCNSIH

>BU_LTP #7(BQ804322) (LTP Bu-7)

MAAPRGAAVVLAMVLAAMVVAPPATVHAAISCSTVYSTLMPCLQYVQQGGSPARGCCTGIQNLLAEANNSPDRRTICGCLKNVANGASGGPYITRAAALPSKCNVALPYKISPSVDCNSIH

>BU_LTP #8(BQ805085) (LTP Bu-8)

MAAPRGAALVLAMVLAAMVVAPPATVHAISCSTVYSTLMPCLQYVQQGGSPARGCCTGIQNLLAEANNSPDRRTICGCLKNVANGASGGPYITRAAALPSKCNVALPYKISPSVDCNSIH

>BU_LTP #9(BQ839001) (LTP Bu-9)

ARRAHAAATLALLVAALVLSAAPAPAEGAVANCGQVVSYLAPCISYAMGRVSAPGGGCCSGVRGLNAAAATPADRKTTCTCLKQQASGIGGIKPNLVAGIPGKCGVNIPYAISQGTDCSKVR

>BU_LTP #10(BQ804802) (LTP Bu-10)

MAARRAHAAAALALLVAALVLSAAPAPAEGAVANCGQVVSYLAPCISYAMGRVSVPGGGCCSGVRGLNAAAATPADRKTTCTCLKQQASGMGGIKPNLVAGIPGKCGVNIPYAISQGTDCSKVR

>BU_LTP #11(BQ807269) (LTP Bu-11)

MARVALVAVFAVLAALAVAEMASGAVTCGDVTSAVAPCMSYARGQASAPSGACCSGVRTLNAKASTPADRKAACNCLKNLAGSGISMGNAANIPGKCGVSVSFPINTKTNCNNLH

>BU_LTP #12(BQ807211) (LTP Bu-12)

MARVALLAVFAVLAALAVAEMASGAVTCGDVTSAVAPCMSYATGQASAPSAGCCSGVRTLNAKASTSADRQAACRCLKKLAGSGISMGNAANIPGKCGVSVSFPINTKVDCNTYILHP

>BU_LTP #13(BQ806247) (LTP Bu-13)

MARAAATQLVLVALVAAMLLVATDAAISCGQVSSALSPCISYARGNGANPTAACCSGVRSLAGAARSTADKQAACKCIKSAAGGLNAGKAAGIPSKCGVSVPYAISASVDCSKIR

>BU_LTP #14(BQ804683) (LTP Bu-14)

MAPAGTRGARAAAPAAVLLVAALLVALALAPRGTSAALTCSTVYNELMPCLGYVQSGGAVPRACCSGIKTLVSRARATPDRRAACACLKTVAAAAAGGPYLGRAAGLPGRCGVQPPFKIDPNVNCNAV

>BU_Peak_4_Protein#1 (farinin Bu-1)

MKTMFILALLALAASTAIAQLETICSQGFGQCQHHQQLGQQQLLDQMKPCVAFVQHQCSPVRTPFPQTRGEQHSSCQTVQHQCCRQLVQIPEQARCKAIQSVEEAIIQQQPQQQWNEPQQEAHLKSMRMSLQTLPSMCNIYVPVQCQQQQQLGRQQQQQLQEQLKPCATFLQHQCRPMTVPFPHTPVQKPTSCQNVQSQCCRQLAQIPEQFRCQAIHNVVESIRQQQHHQPQQEVQLEGLRMSLHTLPSMCKIYIPVQCPATTTTPYSITMTASYTDGTC

>BU_Peak_4_Protein#2(farinin Bu-2)

MKVFILALLALAATTAIAQLETTCSQGFGQSQQQQQPGQRQLLEQMKPCVAFLQQKCSPLRMPFLQTQVEQLSSCQIVQYQCCQQLAQIPERTRCHAIHIVVEAIIQQQSQQQWQEPQQQAQHKSMRMLLENLSLMCNIYVPVQCQQQQQLGQQQQQQLQEQLTPCTTFLQQQCSPVTVPFPQIPVDQPTSCQNVQHQCCRQLSQIPEQFRCQAIHNVAEAIRQQQPQQQWQGMYQPQQPAQLESIRMSLQALRSMCSIYIPVQCPAPTTYNIPLVATYTGGAC

>BU_Peak_4_Protein#3(farinin Bu-3)

MKVFILALLALTATTAIAQLETTCSQGFGQYQQQQQPGQRQLLEQMKPCVAFLQQQCRPLRMPFLQTQVEQLSSCQIVQHQCCQQLAQIPERIRCHAIHSVVEAIMQQQSQQQWQERQQQAQHKSMRMLLENLSLMCNIYVPVQCQQQQQMGQQQQQQQLQEQLTPCATFLQHQCSPVTVPFPQIPVDQPTSCQNVQHQCCRQLSQIPEQFRCQAIHNVAEAIRQQQPQQQWQGMYQPQQPAQHESIRMSLQALRSMCNIYIPVQCPAPTAYNIPMVATCTSGAC

>BU_Triticin#1(BQ804884)(triticin Bu-1)

MAATSFASLSFYFCILLLCHSSMAQLFGMSFNPWQSSRQGGFRECTFNRLQASTPLRQVRSQAGLTEYFDEENEQFRCTGVFAIRRVIEPRGYLLPRYHNTHGLVYIIQGSGFAGLSFPGCPETFQKQFQKYGQSQSVQGQSQSQKFKDEHQKVHRFRQGDVIALPAGIVHWFYNDGDAPIVAIYVFDVNNYANQLEPRHKEFLFAGNYRSSQLHS

>BU_Triticin#2(BQ806424) (triticin Bu-2)

MAATSFASLSFYFCILLLCHSSMAQLFGMSFNPWQSSRQGGFRECTFNRLQASTPLRQVRSQAGLTEYFDEENEQFRCTGVFAIRRVIEPRGYLLPRYHNTHGLVYIIQGSGFAGLSFPGCPETFQKQFQKYGQSQSVQGQSQSQKFKDEHQKVHRFRQGDVIALPAGIVHWFYNDGDAPIVAIYVFDVNNYANQLEPRHKGILCSLATIGVRNFTLVKTYSVGFDVRLLAEALGTSGKIAQRLQSQNDDII

>BU_Triticin#3(BQ804747) (triticin Bu-3)

MAATSFASLSFYFCILLLCHSSMAQLFGMSFNPWQSSRQGGFRECTFNRLQASTPLRQVRSQAGLTEYFDEENEQFRCTGVFAIRRVIEPRGYLLPRYHNTHGLVYIIQGSGFAGLSFPGCPETFQKQFQKYGQSQSVQGQSQSQKFKDRHQKVHRFRQGDVIALPAGIVHWFYNDGDAPIVAIYVFDVNNYANQLEPRHKEFLFAGNYRSSQLHSSQNIFSGFPCSIAC

>BU_Triticin#4(BQ806311) (triticin Bu-4)

DSARGNSQEEQPQMGQSQGEQPQTGQSQGKHIQGEQPQMGQSQAKHYQGDQPEEGQEGQSQEEQSQAGPYPGCQPRAGQSHASQSTYGGWNGLEENFCDHKLSVNIDDPSRADIYNPRAGTITRLNTQTFPILNIVQMSATRVHLYQNAIISPLWNINAHSVMYMIQGHIWVQVVNDHGRNVYNGLLSPGQLLIIPQNYVVMKKAQRDGSKYIEFKTNANSMVSHIAGKSSILGALPVDVIANAYGISRTEA

>BU_Triticin#5(BQ806681) (triticin Bu-5)

EGQSQAKHSQEEQPQMGQSQGEQPQTGQSQGKHIQGEQPQMGQSQAKHYQGDQPEEGQEGQSQEEQSQAGPYPGCQPRAGQSHASQSTYGGWNGLEENFCDHKLSVNIDDPSRADIYNPRAGTITRLNTQTFPILNIVQMSATRVHLYQNAIISPLWNINAHSVMYMIQGHIWVQVVNDHGRNVYNGLLSPGQLLIIPQNYVVMKKAQRDGSKYIEFKTNANSMVSHIAGKSSILGALPVDVIAHAYGIS

>BU_Triticin#6(BQ806874C) (triticin Bu-6)

PILNIVQMSATRVHLYQNAIISPLWNINAHSVMYMIQGHIWVQVVNDHGRNVFNDLLSPGQLLIIPQNYVVLKKAQRDGSKYIEFKTNANSMVSHIAGKNSILGALPVDVIANAYGISRTEARSLKFSREEELGVFAPKFSQSIFRSFPNGEEESS

>BU_Wheatwin#1(BQ806289)(wheatwin Bu-1)

MAVRLMLVAALLCAAAAAAAAQQANNVRATYHYYRPAQNNWDLGAPAVSAYCATWDASKPLSWRSKYGWTAFCGPAGAHGQAACGKCLRVTNPATGAQVTARIVDQCANGGLDLDWDTVFTKIDTNGVGYQQGHLNVNYQFVDCRD

>BU_Wheatwin#2(BQ804430) (wheatwin Bu-2)

MAARLMLVAALLCAAAAAATAQQATNVRATYHYYRPAQNNWDLGAPAVSAYCATWDASKPLSWRSKYGWTAFCGPAGAHGQAACGKCLRVTNPATGAQITARIVDQCANGGLDLDWDTVFTKIDTNGIGYQQGHLNVNYQFVDCRD

>BU_Wheatwin#3(BQ807250) (wheatwin Bu-3)

MAARPMLVVALLCAAAAAATAQQATNVRATYHYYRPAQNNWDLGAPAVSAYCATWDASKPLSWRSKYGWTAFCGPAGAHGQASCGKCLQVTNPATGAQITARIVDQCANGGLDLDWDTVFTKIDTNGIGYQQGHLNVNYQFVDCRD

>BU_Globulin-2_Contig17295 (globulin-2-17295)

MKSTVVRSPWLALALVLSLCLSLSFASWDAEDVGRGSRRWQEGGDDEGRSGSGSGRPYHFGEESFREWAKSRHGHFKVLERFDHELLRGSIGDYRVACLDAAPRAFLQPSHYDADEIAFVREGEGVLVLLRNGKRESFCVREGDVFVIPAGSIVYSANTHRSKWFRVVMLLNPVSTPGSFQEFSPIGFGGEQPQSFFSVFSDEVIQAAFNTRQREDVDRVFQRKSRGEGPISEGSEEQIRELSRSCSRGGRGGGGGSGSEKEDIQPRSLTGEKPRYSNKHGRFHQITGDQCHHLRKLDMDVTLVNITRGSMTALRYATRSTRIYIVVEGRDGYFEMACPHVSSFGRSERREHEQEREREHGHGRRSEEREREHGQGRRSEERKDEQGRQEEEQGRGQEQEKSRGYRQVRAQIKVGSVIVLPAGHPATFVAGNEGNLALLSFGVGANNDEEVFVTGGNSVLKQLDEAAKALAFPQQARELADRVIRAQPESVFVPGPQQQRRVADM

>BU_Globulin-2_Contig 17366(globulin-2-17366)

MKSTVVRSPWLALALVLSLCLSLSFASWDAEDEGRGSRRWQEGGDERRSGESGRPYHFGEESFREWAKSRHGHFKVLERFDHELLRGSIGDYRVACLDAAPRAFLQPSHYDADEIAFVREGEGVLVLLRNGKRESFCVREGDVFVIPAGSIVYSANTHRSKWFRVVMLLNPVSTPGSFQEFSPIGFGGEQPQSFFSVFSDEVIRAAFNTRQREDVDRVFETKSRGEGQISEGSEEQIRELSRSCSRGGRGGGGGSGSEKEDIQPRSLTGEKPRYSNKHGRFHQITGDQCHHLRKLDMDVTLVNITRGSMTALRYTTRSTRIYIVVEGRDGYFEMACPHVSSSGRSERREHEQEREREHGHGRRSEERGQEHGRRSEEEEHGHGGEQEKSRGYRQVRAQIKWGR

>BU_Globulin-2_Contig 18428 (globulin-2-18428)

DHHWLVLAIVLSLCLSLSFASWDAEDVGRGSRRWQEGGDEGRSGGSGRPYHFGQESYREWAKSRHGHFKVLERFDHELLRGSIGDYRVAYLDAAPRAFLQPSHHDADEIAFVREGEGVLVLLRNGKRESFCIREGDVIVIPAGSIVYSANTHRSKWLRVVMFINPVSTPGRFQEFFLIGSGDERPQSFLSVFSDEVIQAALNTRREDVDRVFESKSKGEGEIYEASEEQIRELSRSCSRGGRGGGGSGSEKEDIQPRSLTGEKPRYSNKHGRFHQITGDQCHHLRKLDMDVTLVNITRGSMTALKYTTRSTRIYVVVEGRDGYFEMACPHISSSGRSERREHEQEREREHGQGRRSEEREREQGRGRRSEEREQEQGRQEEEQGHGREQEKSRGYRQVRAQIKVGSVIVLPAGHPATFVAGNDGNLALLSFGVGANNDEEVFVTGGNSVLKQLDEAAKALSFPQQARELADRVIRAQPESVFVAGPQQQRRVADM

>BU_Avenin-like-a#1 (avenin-a Bu-1)

MKTMFLLALLAFTATSAVAQLYTTCSQGYGQCQQQPQPQPQPQPQMNTCAAFLQQCSQTPHVQTQMWQASGCQLVRQQCCQPLAQISEQARCQAVCSVAQIIMRQQQGQSFGQPQQQVPVEIMRMVLQTLPLMCRVNIPQYCTTTPCSTITPAIYSIPMTATCAGGAC

>BU_Avenin-like-a#2 (avenin-a Bu-2)

MKTMFILALLAFTATSAVAQLYTTCSQGYGQCQQQPQPQPQMNTCAAFLQQCIQTPYVQSQMWQASSCQLMRQQCCQPLAQISEQARCQAVCSVSQIIMRQQQGQRFGQPQQQQGQSFGQPQQQVPVEIMRMVLQTLPSMCSVNIPQYCTTTPCSTITPAIYSIPMTATCAGGAC

>BU_Avenin-like-a#3 (avenin-a Bu-3)

MKNLFILALLAFTATSAVAQLYTTCSQGYGQCQQQPQPQPQPQPQPQMNTCAAFLQQCSQTAYVQSQMWQASGCQLMRQQCCQPLAQISEQARCQAVCSVAQIIMRQQQGQRFGQPQQQQGQSFGQPQQQVPVEXMGMVLQTLPSMCSVNIPQYCTTTPCSTIAPAIYNIPMTATCAGGAC

>BU_RTAW#1(BQ838917)(purinin Bu-1)

MKKFLVLALIVVAATTTAAEPFTAFRTAWEPHHPSSPEQQPTPQPQEQPVPHQKLNPCRDALLQQCSPVADMSFLRSQVVQHSSCLVMWEQCCQQLKAIPKQSRCEAIHNVVHAIVLQQQQQLVQGISTQPQQQQQQGQQQQGQGSSQPQQQTQLDQGWIAVIGTWVIQTIPAMCDVHVPPYCYTTISPSIDVTTGMGGY

>BU_RTAW#2(BQ804660) (purinin Bu-2)

MKKFLVLALIVIAATTTAAEPFTAFRIASEPQHPSSPEQQPTLQPQEHPVPHQKLNPCRDALLHQCSPVADMSFLRSQVVQHSSCLVMWEQCCQQLKAIPKQSRCEAIHNVVHAIILQQQKQLVQGTSTQPQQQQQQGQQEHGQGSSQPQHQQQQQQLDQGWIVAIGTWVIQTVPAMCDVHVPPYCYTTISSSSDVTTGMGGY

>BU_RTAW#3(BQ804472) (purinin Bu-3)

MKKFLVLALIVVAATTTAAEPFTAFRSAWEPQHPSSPEHQPTPQPQEHPVPHQKLNPCRDALLQQCSPVADMSFLRSQVVQHSSCLVMWEQCCQQLKAIPKQSRCEAIHNVVHAIILQQQQQLVQATSTQPQQQQQQGQQQQQGLGSSQPQQQTQLDQGWIAVIGTWVIQTIPAMCDVHVPPYCYTTISPSSDVTTDMGGY
