# Supplementary material for: Deciphering the complexities of the wheat flour proteome using quantitative two-dimensional electrophoresis, three proteases and tandem mass spectrometry
Source: Proteome Sci. 2011 Feb 11;9:10. doi: 10.1186/1477-5956-9-10 (PMC3238214; doi:10.1186/1477-5956-9-10)
Supplement: Additional file 3 — Word document listing additional Butte 86 contig sequences not in the Superwheat database but used for subsequent manual evaluation. [file 1477-5956-9-10-S3.DOC]

Additional file 3. Additional protein sequences deduced from Butte 86 contigs that were not included in the SuperWheat database but matched peptides selected by Scaffold.

alpha-gliadin Bu-27

MKTFLILALVATTATTAVRVPVPQLQPKNPSQQQPQEQVPLVQQQQFPGQQQQFPPQQPYPQPQPFPSQQPYLQLQPFPQPQPFLPQLPYPQPQSFPPQQPYPQQRPKYLQPQQPISQQQAQQQQQQQQQQQQQQQQILQQILQQQLIPCRDVVLQQHNIAHASSQVLQQSTYQLLQQLCCQQLLQIPEQSRCQAIHNVVHAIIMHQQEQQQQLQQQQQQQLQQQQQQQQQQQQPSSQVSFQQPQQQYPSSQVSFQPSQLNPQAQGSVQPQQLPQFAEIRNLALQTLPAMCNVYIPPHCSTTIAPFGIFGTN

alpha-gliadin Bu-BQ807130

FGTRSPVPQLQPQNPSQQQPQEQVPLVQQQQFPGQQQQFPPQQPYPQPQPFPSQQSYPQPQPQYPQPQQPISQQQAQLQQQQQQQQQQILQQILQQQLIPCRDVVLQQPNIAHASSQVSQQSYQLLQQLCCQQLWQTPEQSRCQAIHNVIHAIILHHQQQQQQQQQQQQQQQQQQQQQQQQQQQQQPSSQVSYQQPQQQYPSGQGSFQPSQQNPQAQGFVQPQQLPQFEEIRNLALQTLPAMCNVYIPPYCSTTIAP

alpha-gliadin Bu-BQ806209

MKTFLILALLAIVATTATIAVRVPVPQLQPQNPSQQQPQEQVPLVQQQQFPGQQQPFPPQQPYPQPQPFPSQQPYLQLQPFPQPQLPYPQPQLPYPQPQPFRPQQPYPQPQPQYSQPQQPISQQQQQQQQQILQQILQQQLIPCRDVVLQQHNIAHGRSQVLQQSTYQLVQQLCCQQLWQIPEQSRCQAIHNVVHAIILHHHQQQQQQQQPLTQVSFQQPQQQYP

alpha-gliadin Bu-BQ805841

MKTFLILALLAIVATTATTAVRVPVPQLQPQNPSQQQPQEQVPLVQQQQFLGQQQPFPPQQPYPQPQPFPSQQPYLQLQPFPQPQLPYSQPQPFRPQQPYPQPQPQYSQLQQPISQQQQQQQQQQQQQQQQQQQQQQQQQQQQQQQQQQEQQILQQILQQQLIPCMDVVLQQHNIAHGRSQVLQQSTYQLLQELCCQHLWQIPEQSQCQAIHNVVHAIILHQQQKQQQQ

alpha-gliadin Bu-BQ807194

MKTFLILALLAIVATTTTTAVRVPVPQLQPQNPSQQQPQEQVPLVQQQQFLGQQQQQFPGQQQPFPPQQPYPQPQPFLPQLPYPQPQPFPPQQSYPQPQPQYPQPQQPISQQQAQLQQQQQQQQQQQQQQQQQQQILQQILQQQLIPCRDVVLQQPNIAHASSQVSQQSYQLLQQLCCQQLWQTPEQSRCQAIHNVIHAIILHQQQQQQQQQQQQQQQQLQQPSSQVSYQQPQQQYPS

alpha-gliadin Bu-BQ838853

RSQVLQQSTYQLLQELCCQHLWQIPEQSQCQAIHNVVHAIILHQQQKPQQQPSSQVSFQQPLQQYPLGQGSFRPSQQNPQARGSVQPQQLPQFEEIRNLALQTLPAMCNVYIPPYCTIAPFGIFGTN

serpin Bu-1

MATTLATDVRLSIAHQTRFALRLASTISSNPKSAASNAAFSPVSLHSALSLLAAGAGSATRDQLVATLGTGEVEGLHALAEQVVQFVLADASSAGGPRVAFANGVFVDASLLLKPSFQELAVCKYKAETQSVDFQTKAAEVTTQVNSWVEKVTSGRIKNILPSGSVDNTTKLVLANALYFKGAWTDQFDSYGTKNDYFYLLDGSSVQTPFMSSMDDDQYISSSDGLKVLKLP

serpin Bu-2

MATTLATDVRLSIAHQTRFALRLASTISSNPKSAASNAVFSPVSLHVALSLLAAGAGSATRDQLVTTLGTGEVEGLHALAEQVVQFVLADASSAGGPHVAFANGVFVDASLLLKPSFQELAVCKYKAETQSVDFQTKAAEVATQVNSWVEKVTSGRIKDILPSGSVDNTTKLVLANALYFKGAWTDQFDSSGTKNDYFYLPDGSSVQTPFMSSMDDQYLSSSDGLKVLKLPYKQGGDKRQFSMYILLPEAPGGLSNLAEKLSAEPDFLERHIPRQRVALRQFKLPKFKISFETEASDLLKCLGLQLPFSNEADFSEMVDSPMAHGLRVSSVFHQAFVEVNEQGTEAAASTAIKMALLQARPPSVMDFIADHPFLFLLREDISGVVLFMGHVVNPLLSS

serpin Bu-3

MATTLATDVRLSIAHQTRFALRLASTISSNPKSAASNAAFSPVSLYSALSLLAAGAGSATRDQLVATLGTGKVEGLHALAEQVVQFVLADASSTGGPRVAFANGVFVDASLLLKPSFQEIAVCKYKAETQSVDFQTKAAEVTTQVNSWVEKVTSGRIKDILPPGSIDNTTKLVLANALYFKGAWTEQFDSYGTKNDYFYLLDGSSVQTPFMSSMDDQYLLSSDGLKVLKLPYKQGGDNRQFFMYILLPEAPGGLSSLAEKLSAEPDFLERHIPRQRVALRQFKLPKFKISFGIGASDLLKCL

serpin Bu-4

RHEDLFLPCPLFQGAWTDQFDSYGTKNDYFYLLDGSSVQTPFMSSMDDDQYISSSDGLKVLKLPYKQGGDNRQFSMYILLPEAPGGLSSLAEKLSAEPDFLERHIPRQRVAIRQFKLPKFKISFGIEASDLLKCLGLQLPFSDEADFSEMVDSPMPQGLRVSSVFHQAFVEVNEQGTEAAASTAIKMVPQQARPPSVMDFIADHPFLFLLREDISGVVLFMGHVVNPLLSS

serpin Bu-5

MATTLATDVRLSIAHQTRFAFRLASAISSNPESTVNNAAFSPVSLHVALSLITAGAGGATRNQLAATLGEGEVEGLHALAEQVVQFVLADASNIGGPRVAFANGVFVDASLQLKPSFQELAVCKYKAEAQSVDFQTKAAEVTAQVNSWVEKVTTGLIKDILPAGSIDNTTRLVLGNALYFKGAWTDQFDPRATQSDDFYLLDGSSIQTPFMYSSEEQYISSSDGLKVLKLPYKQGGDKRQFSMYILL

serpin Bu-6

APHLIHPKSAASNAAFSPVSLHSALSLLAAGAGSATRDQLVATLGTGEVEGLHALAEQVVQFVLADASSYCSPPVWGCAAVGGGEARASRGEPREERRTEEGNGGVDLRGPRGGGRAGRRHRNAGLRRRAAARARALQRRHID

serpin Bu-7

MATTLATDVCLSVAHQTRFALRLASAISSDPESATGNVAFSPVSLHVALSLITAGAGGTTRDQLVAILGNENAGGPEGLHSLAEQVVQLVLADASITGDPRVAFANGVFVDASLSLKPSFQELAVCNYKSEVQSVDFQNKAPEIASQVNSWVENVTTGLIREILPEGSIDYTTRLVLGNALYFKGLWTEKFDESKTKYDKFHLLNGNTVQTPFMSSTNKQYIS

serpin Bu-8

MKRHIPRQRVALRQFKLPKFKISFETEASDLLKCLGLQLPFSNEADFSEMVDSPMAHGLRVSSVFHQAFVEVNEQGTEAAASTAIKMALLQARPPSVMDFIADHPFLFLLREDISGV
